# Body-resonance: transmission line-like wireless links enabling high-speed wearable communication

**DOI:** 10.1038/s44172-025-00533-z

**Published:** 2025-12-20

**Authors:** Samyadip Sarkar, Qi Huang, Sarthak Antal, Mayukh Nath, Shreyas Sen

**Affiliations:** Elmore Family School of Electrical and Computer Engineering, Purdue University, West Lafayette, IN USA

**Keywords:** Electrical and electronic engineering, Biomedical engineering

## Abstract

Seamless interaction between humans and Artificial Intelligence-empowered, battery-operated, miniaturized devices is reshaping wearable technology by forming an anthropomorphic artificial nervous system that demands high-speed, low-power connectivity. Besides being radiative, radio frequency links suffer absorption losses in non-line-of-sight scenarios and consume more than tens of milliwatts of power. Electro-quasistatic human body communication provides non-radiative links with  ~100X better energy efficiency and  ~30X superior signal confinement over radio wave-based wireless. However, it is limited by  ~60–70 dB path loss, limited bandwidth, and data rates ≤30 Mbps, insufficient for applications such as High definition streaming, and distributed computing at wearable sensor nodes. To overcome these challenges, we propose Body-Resonance Human Body Communication, which leverages the human body’s transmission-line behavior in the near-intermediate field to enhance channel capacity by up to 30X. It achieves approximately 20 dB higher channel gain and a wider bandwidth compared to electro-quasistatic regime, supporting data rates of hundreds of Mbps. Experimental results validate low-loss (~40–50 dB), wideband body channels that are more than 10X less leaky than antenna-based wireless links. Body Resonance can potentially open up the possibilities of immersive augmented/virtual reality and cooperative on-body computing by enabling energy-efficient, high-speed wearable networks across healthcare, defense, and consumer electronics.

## Abstract (web summary)

Conventional body-coupled wireless communication links for wearables rely on electro-quasistatic conduction, treating the human body as a wire. Samyadip Sarkar and colleagues demonstrate leveraging fields beyond electro -quasistatic frequencies unveils the human body’s transmission-line behaviour that enhances wearable-to-wearable communication channel capacity.

## Introduction

**Fig. 1:**
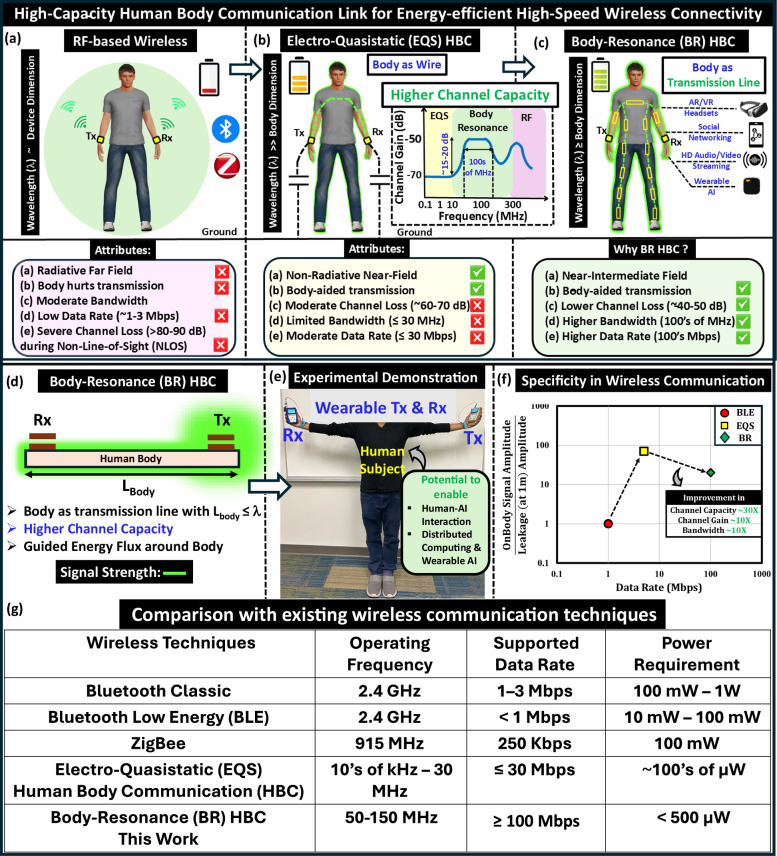
Need for energy-efficient high-speed wireless connectivity for wearable devices. **a** Traditional radio frequency (RF)-based communication: radiative with higher power consumption makes it energy in-efficient (~10 nJ/bit), and besides incurring higher path loss during Non-Line-of-Sight scenarios, it can support a data rate of ≤3 Mbps. **b** Electro-Quasistatic Human Body Communication (EQS HBC): Body as Lossy Wire: non-radiative, hence energy-efficient (~sub-10 pJ/bit) though suffers from moderate end-to-end path loss and limited data rate (≤30 Mbps) due to constraint on its frequency of operation (≤30 MHz) that restricts its bandwidth. **c** Body-Resonance Human Body Communication (BR HBC): Body as Lossy Transmission Line:  ~15–20 dB higher channel gain up to  ~10X wider bandwidth makes its Shannon channel capacity  ~30X higher compared to EQS. It has potential to support high data rate applications ranging from high-definition audio/video streaming to distributed computing with wearable AI. **d** Body as a single-wire transmission line that couples electromagnetic wave onto a cylindrical conductor. **e** Experimental Demonstration of BR HBC system. **f** Communication Specificity: BR HBC offering high data rate while keeping on-body signal order of magnitude higher than off-body leakage. **g** Comparison of the BR HBC with the existing techniques for wireless communication. The human figures are made in Open-source software: `MakeHuman'.

The continuous growth of miniaturized computing and communication technologies has revolutionized the cooperation between humans and electronic devices. The increase in the use of such battery-operated smart devices in consumer and medical electronics, remaining a crucial part of the Internet of Things (IoT)^1–4^, calls for energy-efficient wireless interconnectivity for long-term reliable operation. Moreover, devices such as smartphones, smartwatches, smart glasses, AI Pocket Assistants, and AR/VR headsets, while becoming ubiquitous, create a network of wireless devices around our body known as the Internet of Bodies (IoB)^5–8^. With an approximate market share of 10–15% in the electronics industry, these wearables hold significant potential for developing an anthropomorphic artificial nervous system architecture that could transform both the wearable technology and bio-electronics sectors, opening up possibilities for innovative applications and enhanced user experiences. To support high-speed connectivity at low-power yet perpetual operation demand, an architectural shift from the state-of-the-art techniques of wireless communication, as illustrated in Fig. 1.

Owing to their radiative nature, the traditional radio frequency (RF)-based wireless communication methods among these devices consume higher power (>10s of mW) and experience severe transmission losses (~80–90 dB or even higher) from the absorption from the body and surroundings^9,10^ in non-line-of-sight scenarios, making them less suitable for IoB applications that demand superior energy efficiency and physical security. Besides, their limited maximum data rates (≤3 Mbps) restrict their scope of application, depicted in Fig. 1a.

Addressing the need for energy-efficient connectivity, Human Body Communication (HBC) leverages the electrically conducting properties of the human body to facilitate energy-efficient information exchange among wearable devices. The exploration of different modalities of HBC over the last couple of decades has unveiled enabling data transfer via electric fields^11–15^, magnetic fields^16–18^, and acoustics^19–21^, etc. Being transparent to the quasistatic magnetic field (frequency ≤ 30 MHz), the presence of the human body doesn’t influence the channel behavior when the signal transmission occurs via the means of the magnetic field. Moreover, the inductive links, being susceptible to the relative separation and the orientation mismatch between the transmitter and receiver coils, cause sharper attenuation in transmission channel gain that makes magneto-quasistatic HBC not an optimal choice for long-range on-body channels^16,17,22^. Furthermore, the body tissues, being conductive, attenuate the propagating high-frequency Magnetic HBC fields because the induced eddy currents hinder the efficient propagation of waveguide modes within the body, making it not a preferred choice for high-speed body-communication links^18^. Moreover, the limited coverage area and inability to support higher data rate communication restricts the application scope for transmitting information via acoustic signals through the human body^19–21,23^.

Among the different modes of electric HBC, the galvanic HBC^24^ has limited coverage due to the dipole-dipole interaction governing its operation, which reduces the channel gain away from the transmitter. On the contrary, the capacitive HBC operating in the Electro-Quasistatic (EQS) frequency regime emerged as a promising choice for its frequency-independent approximately consistent channel gain across the entire body channel with high-impedance capacitive termination at the receiver^13^. This makes capacitive EQS HBC suitable for on-body communication with higher link coverage. In EQS capacitive HBC, the signal gets coupled into the body from a transmitter using single-ended excitation comprising a signal electrode that remains in contact with the body, and the ground electrode of the device is left floating. With its lower carrier frequency (typically ≤30 MHz) resulting in the operating wavelength (≥10 m) being order of magnitude larger than human body dimensions (≤2 m), in capacitive EQS HBC, the human body, remaining at the same potential throughout, constitutes the forward path and the parasitic couplings from the ground of the communicating devices to the earth ground form the return path^25^. Its non-radiative nature, which results in better signal confinement, makes this mode energy efficient (~sub-10 pJ/bit)^14,26^ and physically secure over RF^27^. However, its limited channel capacity ($C=B{\log }_{2}(1+\frac{S}{N})$, according to Shannon Hartley Theorem) due to its lower operating frequency (≤30 MHz) and moderate channel loss (~60–70 dB) becomes a bottleneck as it falls short in supporting high-speed applications requiring 100s of Mbps operational data rate, presented in Fig. 1b.

Addressing these limitations, this paper introduces a modality for body communication, where the human body acts as a lossy transmission-line-like channel that utilizes the electromagnetic resonant phenomena has potential to offer up to a  ~30X improvement in the body channel capacity (i.e.,  ~10X increase in SNR and improvement in operational bandwidth as shown in schematic of body-channel characteristic), illustrated in Fig. 1c. Leveraging the human body as an electrically distributed conductor for information transfer, this mode of communication ensures guided energy-flux around user’s body which is otherwise non-guided with radiative RF-based techniques, illustrated in Fig. 1d. The experimental demonstration of BR HBC with wearable form factor devices is shown in Fig. 1e. Besides, the demand of higher channel capacity, the ratio of on-body to off-body signal strength (i.e., leakage) remains crucial as it functions as a figure of merit for specificity of an wireless link. The variation of the aforementioned ratio with potential data rate is captured in Fig. 1f. A comparative analysis of the proposed BR-HBC, with state-of-the-art techniques for wireless communication (such as Bluetooth, BLE, ZigBee, EQS) for IoB devices, is presented in Fig. 1g. This approach enables low-loss (~40–50 dB), high-speed (100s of megabits per sec) connectivity for wearable devices that inspire the emergence of various body-centric technologies that include but are not limited to high-definition audio-video streaming, distributed computing with wearable AI, and seamless interaction between human and augmented-virtual reality-based devices.

Previously, very few works attempted to enable high-speed communication modes around the human body for wireless body-area network devices. The key attributes from the prior arts in comparison to the proposed BR HBC are presented in Supplementary Discussion 11^28^. Despite the limited number of studies, none has thoroughly examined the fundamental principles underlying body-resonance communication and provided an in-depth theory to support them. This highlights the need for a foundational, physics-based analysis that connects insights from Field Theory and Circuit Theory. Such a connection could ultimately support the development of energy-efficient transceivers for high-speed, body-centric communication.

Though, due to the increased radiative component from the transmitter and humans, the body channel in the BR frequency range appears to be more leaky than the EQS mode of communication, the crucial need to enhance the channel capacity has prompted us to ask: Can we utilize the electromagnetic waves, resonant to human body’s dimensions to facilitate energy-efficient, high-speed connectivity at speeds of hundreds of megabits per second among BAN devices?

In this work, we demonstrate BR HBC that enables energy-efficient fast connectivity for wearables by utilizing the transmission line-like behavior of the human body, and the contributions of the proposed modality are summarized below:

- Enabling Higher Channel Capacity: This work enables high-capacity body channel (up to  ~30X higher than EQS) that offers low-loss, wide-band high-speed (~100s of Mbps) wireless connectivity that will potentially inspire the design of energy-efficient transceivers and emergence of body-centric applications using wearables that facilitate augmented living.
- Body as a Lossy Transmission Line: Presenting a perspective by conceptualizing the human body as an unbalanced lossy transmission line, we consider the body acts as a signal conductor, while the earth’s ground serves as an additional conductor. These understandings are supported by numerical electromagnetic simulations and experiments, demonstrating the electrically distributed characteristics of the body channel in the BR frequency range.
- Body-channel characterization for reliable communication: With the body being a lossy transmission line, the variability in the body-channel characteristic in the BR frequency regime depends on several factors, influencing the transmission line parameters that remain decisive in optimizing the channel capacity. The body channel is characterized to estimate the capacity of various body-communication links based on the receiver sensitivity and the adequate data rate for reliable communication.
- Leakage profile analysis of the proposed body-communication link: Specificity in information exchange remains crucial while analyzing a wireless communication channel. This work presents the results from the leakage measurements around an on-body communication link and provides a comparative analysis of its signal confinement behavior with traditional radiative communication like Bluetooth.

## Results

The following section depicts the results from numerical simulations and measurements with wearable devices that illustrate the benefits of enabling human body communication in the Body-Resonance frequency regime.

### Numerical simulation results

To support the developed understanding from the theory, as described above, here we present the results of Electromagnetic (EM) simulations performed within a Finite Element Method (FEM) based electromagnetic solver, namely High-Frequency Structure Simulator (HFSS) from ANSYS. Simulations are executed on a simplified cross-cylindrical human body model, whose accuracy has been confirmed previously through comparison of its field and current density distribution with a detailed model by Maity et al.^29^. With an input excitation as an ideal AC voltage source of amplitude 1 V, from the received voltage (*V*_*Rx*_) variations, the channel gain is calculated from the induced electric field.

#### Signal propagation in EQS and above

**Fig. 2:**
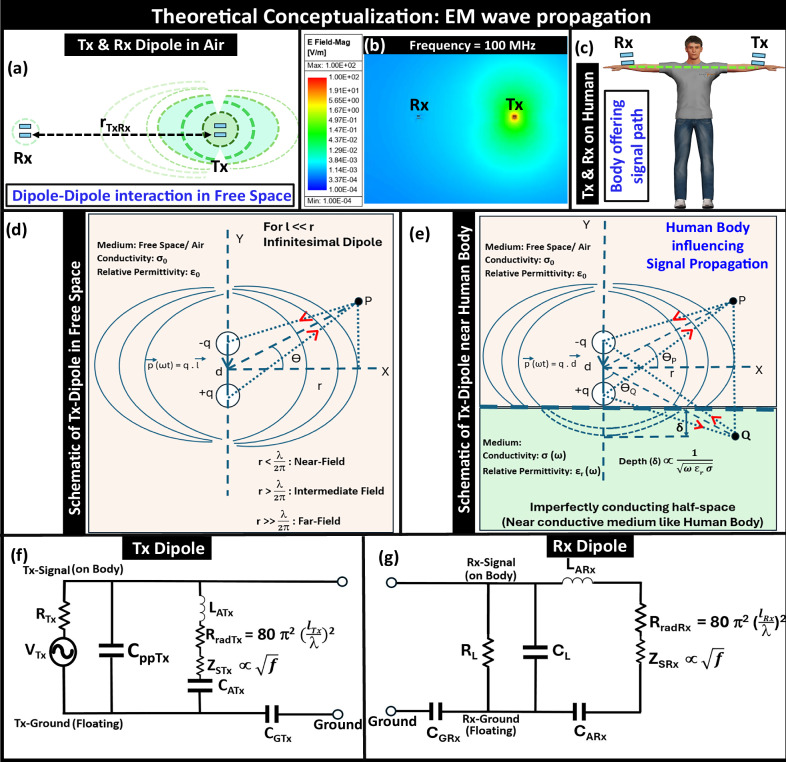
Conceptualizing electromagnetic wave propagation behavior in the presence of the Human Body. **a** Transmitter (Tx) and receiver (Rx) dipole communicating in Air, **b** Electric-field plot of the Tx-Rx interaction at 100 MHz, **c** Human Body Communication with Tx and Rx on the body. Schematic representation of the radiation from Tx-Dipole: (**d**) in Free-space /Air, (**e**) in the presence of the human body. Conceptual Circuit model: (**f**) Tx-dipole, (**g**) Rx-dipole.

Here we delve into the influence of the human body on electromagnetic signal propagation behavior, illustrated in Fig. 2. Aiming to simplify our understanding of the complex nature of EM wave propagation around the body, we start from the radiation theory of a Hertzian dipole while considering our wearable transmitter and receiver as an infinitesimal dipole (under the following assumptions: the size of the transmitter i.e., effective dipole length (l)  << operating wavelength (*λ*)) communicating in the air (i.e., in an infinite space), schematically shown in Fig. 2a. Now, depending on the ratio of the field observation point (r) to *λ*, the propagation region around the Tx dipole can be sub-divided under the following categories: (a) near-field (*βr* < < 1), (b) intermediate field (*βr* > 1), and (c) far-field (*βr* > > 1), where *β* represents the wavevector as schematically presented in Fig. 2d.

With *l* representing the thickness of the transmitting device (i.e., the separation between the signal and the ground plate) and I denoting the current supplied to the signal electrode, *η* standing for medium’s wave impedance, (*r*, *θ*, *ϕ*) standing for the spatial location of the point of observation, the following well-known equations depict the electromagnetic fields from a Hertzian dipole:

1a $${E}_{r}=\eta \frac{{I}_{0}l\cos \theta }{2\pi }\left[\frac{1}{{r}^{2}}+\frac{1}{j\beta {r}^{3}}\right]{e}^{-j\beta r}$$

1b $${E}_{\theta }=j\eta \frac{\beta {I}_{0}l\sin \theta }{4\pi }\left[\frac{1}{r}+\frac{1}{j\beta {r}^{2}}-\frac{1}{({\beta }^{2}{r}^{3})}\right]{e}^{-j\beta r}$$

1c $${E}_{\phi }=0$$

1d $${H}_{r}={H}_{\theta }=0$$

1e $${H}_{\phi }=j\frac{\beta {I}_{0}l\sin \theta }{4\pi }\left[\frac{1}{r}+\frac{1}{j\beta {r}^{2}}\right]{e}^{-j\beta r}$$

In the near-field region, since *βr* < < 1, the term involving $\frac{1}{{r}^{3}}$ becomes dominant. Therefore, at lower frequencies, i.e., in Electro-Quasistatics (EQS) (10s of MHz range or less), where the wavelength of the signals is orders of magnitude larger than the dimensions of the communicating devices and the human body, the dominant mode of transmission is the quasistatic near-field. Hence, the transmitter can be simplified as an electro-quasistatic dipole exciting the human, which can be approximated as a node, as the consistent potential exists throughout the body. Besides, below a certain frequency (*f*_*c*_), the voltage developed by the magnetic fields (*H*_*ϕ*_), associated with an electric dipole, is usually ignored, as there are no closed current loops (i.e., no formation of the magnetic dipole) present at the electrodes of the devices. Now, when the Tx and Rx dipoles are mounted on the human body, the EQS signal from the Tx gets capacitively coupled to the human body and picked up at the Rx. This enables the EQS HBC, which has been broadly explored^13,25,30^. However, as the operating frequency increases beyond the EQS range, the near-field quasistatic approximation of the Tx-dipole no longer holds accurately as its reactive part of the transmitted energy starts influencing signal transmission. Therefore, the influence of the intermediate field also needs to be emphasized. The boundary between the near and the intermediate field can be conceptualized via a distance (r_*d*_) from the source, namely the radiation zone, which can be calculated as, r_*d*_ = $\frac{1}{\beta }$ = $\frac{\lambda }{2\pi }$. While in the intermediate field region, the term $\frac{1}{{r}^{2}}$ predominates, in the far field, the term $\frac{1}{r}$ decides the field decay characteristics. The E-field plot for dipole–dipole interaction in air at 100 MHz is presented in Fig. 2b. The interaction between electric dipoles can be approximately captured in conceptual circuit models, shown in Fig. 2 (f, g), where the inductances (*L*_*ATx*_, *L*_*ARx*_), capacitances (*C*_*ATx*_,  *C*_*ARx*_) and radiation resistance (${{{{\rm{R}}}}}_{radTx},\,{{{{\rm{R}}}}}_{radRx}\,\propto \,\frac{1}{{\lambda }^{2}}$) are used to model the radiative behavior of the Tx and Rx. The circuital branches consisting of the following elements ((*C*_*ppTx*_, *L*_*ATx*_, *C*_*ATx*_, *R*_*radTx*_) at Tx can be combined and simplistically assumed to be an impedance element i.e., *Z*_*ppTx*_ at Tx and similarly (*C*_*ppRx*_, *C*_*ARx*_, *L*_*ARx*_, *R*_*radRx*_) can be combined to *Z*_*ppRx*_ at Rx. Developing a detailed circuit model in this frequency regime of interest would be interesting while remains beyond the scope of this study, will be part of our future work. Although a detailed model is not presented, the simplified impedance element-based model for analyzing the channel performance is presented in Supplementary Discussion 7^28^. These model gets reduced to the models of an EQS capacitive Tx and Rx at lower frequencies (i.e., *f* ≤ 30 MHz).

**Fig. 3:**
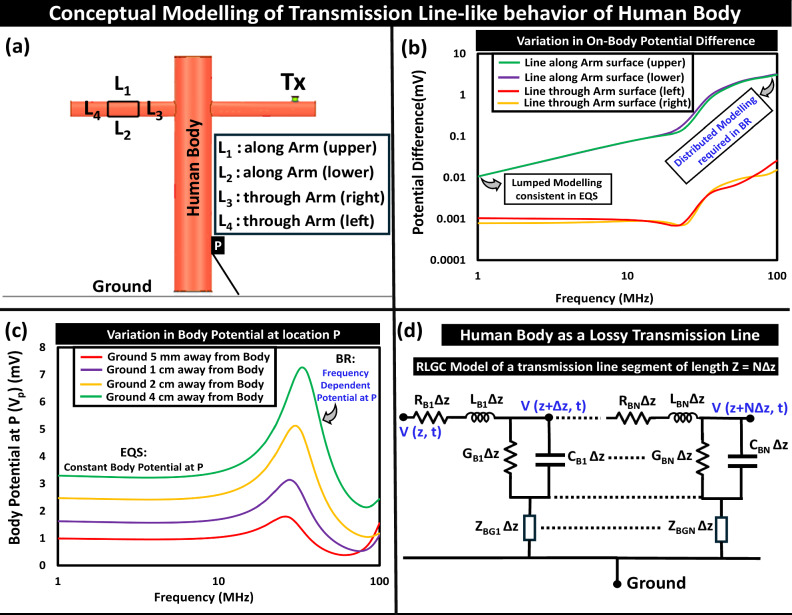
Conceptual modeling of human body as a lossy transmission line. **a** Numerical simulation setup for electromagnetic analysis, **b** Variation in the potential difference between two locations, highlighting the need for distributed modeling of the body in BR, **c** Variation in Body potential at location P relative to the ground, illustrating frequency dependency of potential P, **d** Conceptual RLGC circuit of the human body, modeled as a lossy unbalanced transmission line with the human body being the signal conductor and the earth’s ground as the ground conductor.

With the dominance of the parasitic return path on the estimated path loss with wearable devices, previous studies on capacitive EQS HBC analyzing simplified lumped elements-based biophysical models provided fairly consistent results for the body channel characteristic. However, the increased complexity of the propagation mechanism in the BR frequency regime demands more of a distributed modeling of the human body which can be conceptualized in a unbalanced lossy transmission line-like behavior with the body acting as a signal conductor and the earth’s ground acting as a ground conductor, illustrated in Fig. 3. The increased variation in the potential difference between two locations on the human body with an increase in frequency highlights the requirement for distributed modeling of the body channel in the BR, which can otherwise be regarded as a lumped node in the EQS regime, depicted in Fig. 3b. Moreover, the frequency-dependent potential variability at a point P in BR, delineated in Fig. 3c, presents the influence of the ground location. A conceptual model for the body channel behavior as a lossy transmission line is presented in Fig. 3d. This RLGC-based model can be simplistically regarded as a distributed network of impedance elements (*Z*_*Body*_) i.e., the electrical contribution of (*R*_*Bi*_, *L*_*Bi*_, *C*_*Bi*_, *G*_*Bi*_) can be assumed as an impedance: *Z*_*Body*_ = *R*_*Body*_ + *jX*_*Body*_. The detailed derivation of the channel transfer function is presented in the Supplementary Discussion 7^28^. In very few previous attempts to capture the dispersion and attenuation characteristics of the body channel, a transmission line model (TLM) using infinite structures and a periodic transmission line Model of finite length (over a frequency range from 1 kHz to 1 MHz)^31^, were studied. Although developing a detailed biophysical model capturing the channel variability in the BR regime is beyond the scope of this paper, serves as a motivation for future work. The field around the transmission line-like human body, being a function of the total axial current, changes based on the (i) incident field, (ii) dielectric properties of the body tissues (relative permittivity (*ϵ*_*r*_) and conductivity (*σ*)), (iii) dimensions of the human model (length and width of the transmission line), and (iv) the load impedance from the subject’s body (Z_*B*_) to the earth’s ground. The variation in current density within the body model and the distribution of the H-field around the body are presented in Supplementary Discussion 1^28^. This variation illustrates the electrically distributed, transmission-line-like behavior of the body channel in the BR frequency regime, which contrasts with the wire-like behavior observed in the EQS. Hence, analyzing the influence of these factors is crucial for understanding the body channel behavior.

#### Enabling energy-efficient high-speed connectivity via BR HBC

**Fig. 4:**
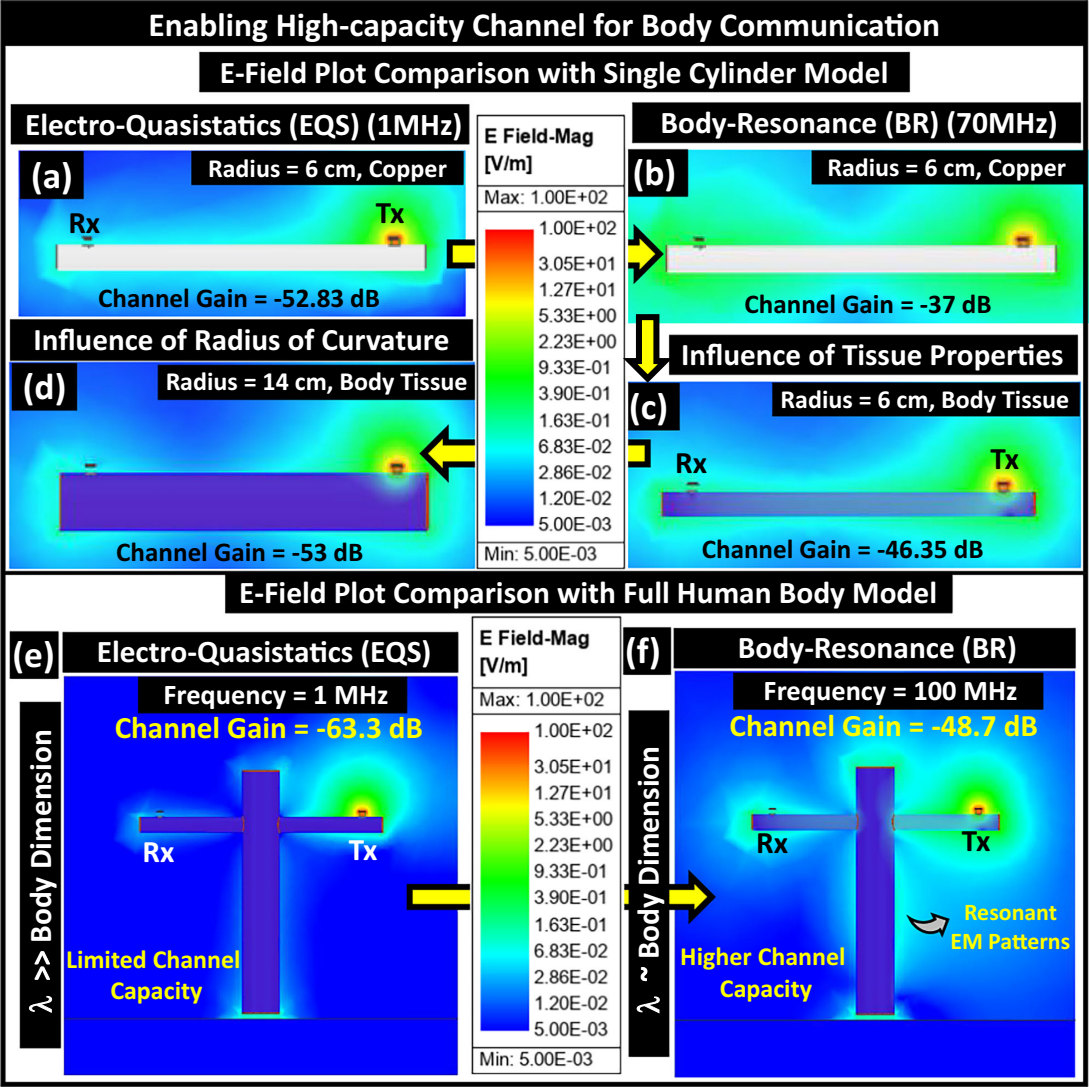
Reductionist approach to analyze electromagnetic resonance of conducting medium. Single-cylinder model: Tx *&* Rx on a copper cylinder, emulating human arm: (**a**) in EQS, (**b**) in BR, Dependency on Tissue Properties: (**c**) cylinder material replaced with body tissue (skin and muscle), Dependency on Limb Thickness: (**d**). The cylinder radius increased to emulate the human torso. Cross-cylindrical Human Body Model: E-Field Plot comparison: (**e**) Lower field strength at Rx indicates limited channel capacity, (**f**) Higher field strength at Rx denotes higher channel gain that results in an improvement in channel capacity.
- Next, the operating frequency is increased to 70 MHz, i.e., to the peak frequency in the transfer characteristic, to observe the effect of the resonance phenomena of the copper cylinder. We notice a  ~15 dB improvement in the simulated channel gain as it becomes −37 dB. The field plot, shown in Fig. 4b, depicts the improvement in E-field strength at the Tx and Rx and the formation of an E-field null in between.
- Subsequently, to investigate the influence of tissue properties on the resonant behavior of the cylindrical resonator, we change the material property of the cylinder to the dielectric properties (conductivity (*σ*) and relative permittivity (*ϵ*_*r*_)) of the body tissues (skin and muscle), adopted from the works of Gabriel et al. We observe a  ~9 dB attenuation in channel gain due to a combination of the following: (i) the higher relative permittivity (*ϵ*_*r*_) of the tissues than air that reduces the propagation velocity (${{{{\rm{v}}}}}_{p}\,\propto \,\frac{1}{\sqrt{{\epsilon }_{r}}}$) and (ii) the conductivity (*σ*) of the body tissues that is several orders of magnitude lower than copper. Besides, the E-field plot, presented in Fig. 4c, shows the resonance of the tissue cylinder with a reduced quality factor (*Q*) from the dielectric loss. The reduced *Q* of the human body, being an imperfect resonator, gives rise to a relatively higher loss wide band channel (Bandwidth (BW) = $\frac{{f}_{c}}{Q}$) than a conducting cylinder while reducing its radiation efficiency.
- Furthermore, to study the effect of a change in the limb thickness (i.e., thickness of transmission line), in other words, the radius of curvature of the body cylinder, we increase the cylinder radius to 14 cm to mimic the lateral dimension of the human torso. An attenuation in channel gain is observable as it goes down to −53 dB due to higher path loss from the increased tissue thickness. The field plot highlights the reduced magnitude of the E-field strength at the Rx in Fig. 4d.
- Finally, a comparative analysis of the field plot with the entire cross-cylindrical human body model between EQS (at 1 MHz) and BR (at 100 MHz) is provided in Fig. 4e, f. Though offering better signal confinement with its non-radiative behavior, EQS HBC limits the application’s data rate with its lower bandwidth. The increased E-field strength around the body highlights the benefits of enabling HBC in the BR frequency regime, as it supports a high-capacity channel.

To investigate the influence of the aforesaid factors on channel capacity, we take a reductionist approach. This section discusses the potential for using the human body as a high-capacity channel for wireless communication between transmitters (Tx) and receivers (Rx). Specifically, we analyze a single cylindrical model that is 180 cm long, equivalent to the height of a human, before progressing to a full-body model as follows:

- We began by considering a scenario where the Tx and Rx, separated by a distance of 150 cm, are mounted on a copper cylinder with a 6 cm radius, similar to the dimensions of a human arm. After numerically simulating the single-cylinder model for electromagnetic analysis in ANSYS HFSS at a frequency of 1 MHz, the simulated channel gain is determined to be −52.83 dB. The electric field plot, depicted in Fig. 4a, illustrates the confinement of the transmitted signal around the cylinder.

#### Human body influencing signal transmission

**Fig. 5:**
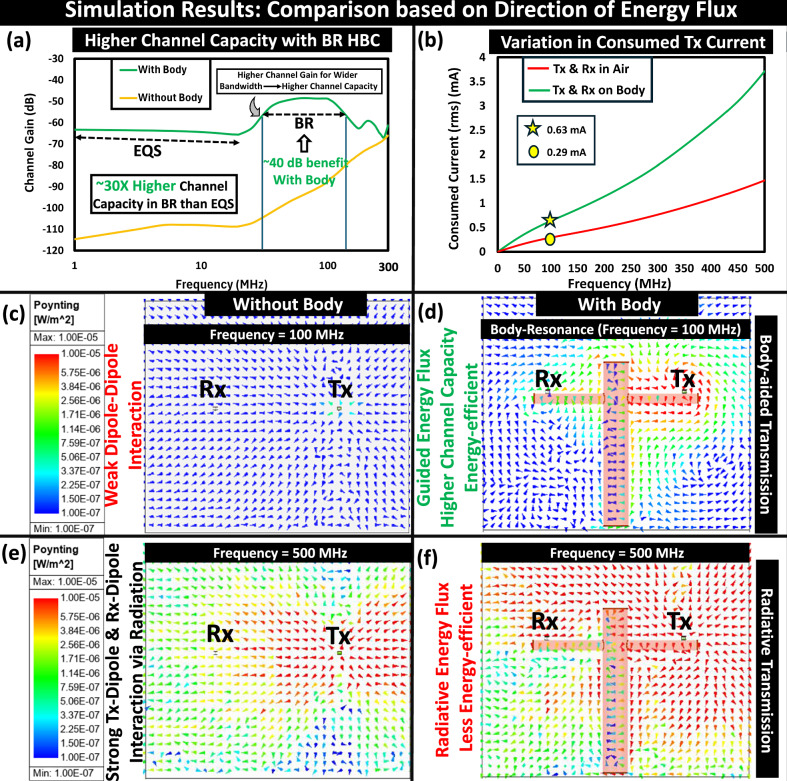
Human body as a energy-efficient high-speed communication channel in body-resonance (BR). **a** Human body providing higher bandwidth and  ~20 dB higher channel gain that leads to up to a  ~30X improvement in body channel capacity over Electro-quasistatic (EQS) regime. **b** Transmitter (Tx) current variation depicting the need for careful choice for operational frequency range that optimizes the trade-off between energy efficiencies and faster connectivity. **c** In the absence of humans, the Tx and receiver (Rx) dipoles weakly interact via dipole-dipole interaction, **d** Human body supporting guided energy flux density while improving channel capacity in BR, **e** Stronger Tx-Rx interaction exists via radiation at a higher frequency, **f** Body acting as an extended ground boosts the radiation from Tx, resulting in non-guided energy flux density.

In the absence of the human body, when the devices (Tx *&* Rx) wirelessly communicate in air, the dipole-dipole interaction between Tx and Rx decides the received signal strength, i.e., the coupled field strength in EQS and radiated field strength when beyond EQS (in the EM frequency regime). However, with its electrically conducting nature, the presence of the human body offers a conducting path for signal transmission during the on-body positioning of the Tx and Rx and decisively influences the received signal strength. The body channel characteristic, presented in Fig. 5a, depicts the benefits of enabling BR HBC as it offers  ~40 dB higher channel gain compared to device-to-device coupling (i.e., when the devices wirelessly couple through the air). Besides offering higher channel gain, being a conductor of conductivity (*σ* ~ 0.6–0.7 S/m) several orders of magnitude smaller than metals, the human body supports wider operational bandwidth (due to its lower quality factor resulting from the Body-Resonance), which enables high channel capacity. The location of the peak in the channel characteristic between  ~50–150 MHz confirms its appearance from the resonance phenomena of the human body since the devices are small enough (i.e., ≤3 cm) to introduce peaks from their antenna characteristics (i.e., at 2.5 GHz if quarter-wave dipole and 5 GHz if half-wave dipole in air) in the channel behavior in BR frequency regime. Consequently, when it comes to iso-sensitivity at the receiver (assuming −90 dBm), theoretically signal-to-noise ratio (SNR) can be improved by a factor of 10, while also increasing the data rate by 5X from EQS-HBC to BR-HBC. However, the variation in the consumed transmitter current, portrayed in Fig. 5b, depicts that in an attempt to enable a high data rate, the human body starts drawing more current from the transmitter in the BR regime, which may raise some concerns from the user’s safety viewpoint which demand an analysis of the user’s safety, as per the guidelines issued by the International Commission on Non-Ionizing Radiation Protection (ICNIRP)^32^, and hence addressed in a subsequent subsection. Moreover, the benefits of BR HBC can be visualized via observing the direction of energy flux density, i.e., the Poynting vector plot (*S* = *E* × H^*^), highlighting strong guided energy flux density along the body surface, i.e., directive signal flow that strengthens the field at Rx at BR, illustrated in Fig 5d. However, with a further increase in the operating frequency, the dipole-dipole interaction between Tx *&* Rx in the air starts becoming stronger, shown in Fig. 5e, which gets boosted even further by the presence of the human body, making the energy flux density more non-guided, illustrated in Fig. 5f. This happens due to a combination of the following: (i) a shift in the device peaks to a lower frequency due to the higher dielectric index of the surrounding body tissues (i.e., ${{{{\rm{f}}}}}_{c}\,\propto \,\frac{1}{\sqrt{{\epsilon }_{tissue}}}$), that occurs due to an increase in the effective electrical length of the communicating devices (L/*λ*_*eff*_), (ii) the human body as a large conductor acting as an extended ground to boost the gain of the transmitting and receiving antennas, and (iii) some part of the wave gets guided along the body surface as a surface wave. Though offering higher channel gain, the highly non-guided energy flux density, depicted in Fig. 5f, makes it a radiative transmission and not an energy-efficient choice for the long-term operation of battery-powered wearables. Animation plots, capturing the variation in E-field and Poynting Vector (Energy Flux Density) in frequency domain are included with this manuscript as Supplementary Movie 1^28^. The variation in the magnetic field is illustrated in Supplementary Movie 2^28^, which shows H-loops that encircle the body and return via the ground, confirming the guided transmission line behavior between the body cylinder and the earth’s ground plane. The transient variation in the E-field at a selected BR frequency of 100 MHz is captured in Supplementary Movie 3^28^.

#### Influence of body tissues and limb thickness

**Fig. 6:**
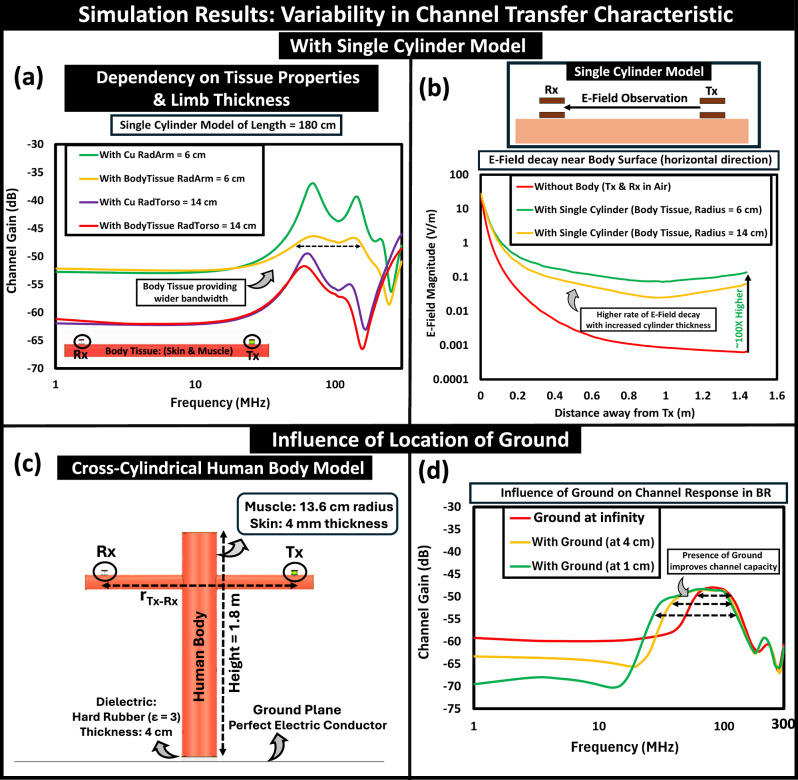
Analysis of channel transfer characteristics: with single cylinder model. **a** Influence of dielectric properties of body tissues and limb thickness on channel gain variability. **b** Electric Field (E-Field) decay profile over distance from the transmitter (Tx) along the body surface, Influence of Ground Location: (**c**) Numerical simulation setup with cross-cylindrical human body model (Front view), **d**. Variation in ground location relative to the subject’s body affects operational bandwidth and channel capacity.

To understand how the tissue properties affect the behavior of the body channel, we conducted an analysis using a single-cylinder model. The transfer characteristic shown in Fig. 6a illustrates the dependence of channel gain on the dielectric properties of tissues (skin and muscle) and compares it with a conducting cylinder made up of copper. While body tissues, as imperfect electrical conductors, provide relatively lower channel gain, they can resonate with a lower quality factor from their inherent dielectric losses, allowing for a broader bandwidth over a conducting cylinder. The influence of tissue properties on channel characteristics of the full human model in the BR regime is analyzed in detail in Supplementary Discussion 8^28^. We also observed that an increase in the radius of the single-cylinder model, mimicking the thickness of a torso, led to a reduction in channel gain due to the thicker cylinder resulting in a sharper rate of attenuation in the observed E-field strength along the surface of the cylinder, as shown in Fig. 6b. Furthermore, there was about a 100-fold improvement in the E-field strength along the body surface compared to device-to-device coupling, validating the previously observed channel gain benefits and providing motivation for analyzing BR-HBC.

#### Location of ground influencing body channel capacity

It is crucial to analyze the subject’s body position on the ground to optimize the body’s signal transmission capacity for capacitive HBC. The numerical simulation setup to capture the change in the ground position about the subject’s body is presented in Fig. 6c. In the context of EQS, being close to the ground reduces the channel gain by influencing the load impedance from the body (Z_*B*_ = $\frac{1}{s{C}_{B}}$) by amplifying the parasitic capacitive coupling (C_*B*_) between the human body and the earth’s ground, as ${{{{\rm{V}}}}}_{out}\,\propto \,\frac{1}{{C}_{B}}$. Additionally, it shifts the zero of the channel transfer function to a lower frequency, and hence, reduces the EQS flat band range, i.e., the operational bandwidth. However, in the BR frequency regime, the human body acts as a lossy resonator, and being closer to the ground causes the transfer function second pole to shift to a higher frequency. This results in an enhancement in operational bandwidth and thereby improvement in channel capacity, as illustrated in Fig. 6d. We also examined the behavioral differences of BR HBC in Machine–Machine (M2M) and Wearable–Wearable (W2W) scenarios. Our analysis depicts that the M2M/M2W setup shifts the BR peak to a lower frequency as the human body behaves like a quarter wave monopole (i.e., ${f}_{r}\propto \frac{1}{4{l}_{Body}}$) in proximity to earth’s ground, which results in reduced channel capacity compared to the W2W setup where the increased return path impedance resulting from the reduced return path capacitance at the Tx and Rx, shift the body resonance peak to a higher frequency. This is discussed in Supplementary Discussion 2^28^.

#### Selection of termination impedance

**Fig. 7:**
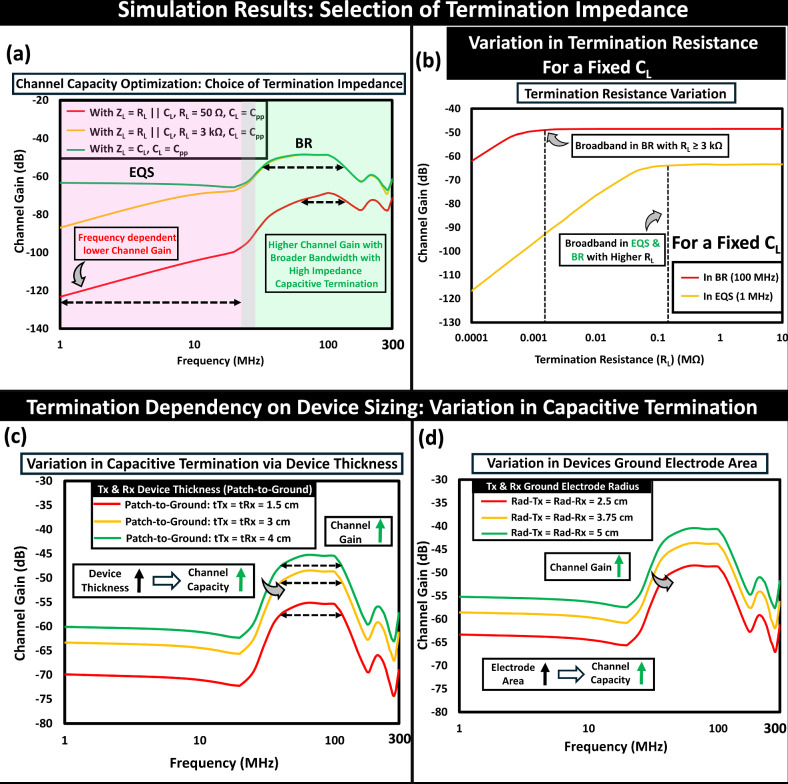
Choice of termination impedance. **a** Variation in channel gain occurs with different types of termination, such as resistive, capacitive, or a combination of both. With a choice of high-impedance capacitive termination, broadband channels ranging from EQS to BR regime can be enabled. **b** Variation in channel gain with a change in termination resistance. Influence of Device Sizing: (**c**) an increase in device thickness reduces the effective termination impedance at the receiver, thereby improving the channel gain. **d** Increasing the ground electrode area of the devices increases the effective return path impedance at the receiver, resulting in an improved channel capacity.

The choice of termination impedance (Z_*L*_) is critical for enhancing the efficiency of high-speed body-centric communication with wearable devices. It has a notable impact on the channel gain and operational bandwidth, as illustrated in Fig. 7a. In voltage mode communication, a system with effective low-impedance resistive termination (Z_*L*_ = *R*_*L*_∥C_*L*_ where *R*_*L*_ = 50 Ω) provides lower channel gain in the EQS regime that varies with frequency at 20 dB/decade. While this system offers relatively higher channel gain for a wider bandwidth in the BR regime compared to EQS, it is not the optimal choice for channel capacity maximization. This system can further improve its channel capacity in the BR regime by using a high impedance termination (i.e., Z_*L*_ = *R*_*L*_∥C_*L*_ where R_*L*_ ≥ 3 kΩ). For a fixed *C*_*L*_ of 2.3 pF, the impact of varying the receiver’s load resistance (*R*_*L*_) at operating frequencies in EQS and BR is depicted in Fig. 7b. However, using a higher resistive load at the receiver will increase the contribution of thermal noise voltage at higher frequencies and eventually degrade the signal-to-noise ratio at the output. Using a high impedance purely capacitive termination (i.e., Z_*L*_ = $\frac{1}{j\omega {C}_{L}}$) at the receiver, channel capacity can be enhanced over a wide-band channel spanning from a few tens of kHz to hundreds of MHz, while also making the channel gain in the EQS regime frequency independent. The size of the communicating devices may also influence the channel capacity as they modify the effective termination reactance (*X*_*c*(*eff*)_) from the parasitic coupling between their ground and the human body. Increasing the thickness of the devices leads to improved channel capacity due to the rise in channel gain resulting from the reduced effective load impedance (Z_*L*(*eff*)_) at Tx and Rx, as shown in Fig. 7c. Moreover, a larger area of the ground electrode of the devices can provide improvement in the channel gain (4X increase in area of the Tx and Rx ground resulting in  ~2.5X increment in channel gain), presented in Fig. 7d.

#### Factors contributing towards channel variability

**Fig. 8:**
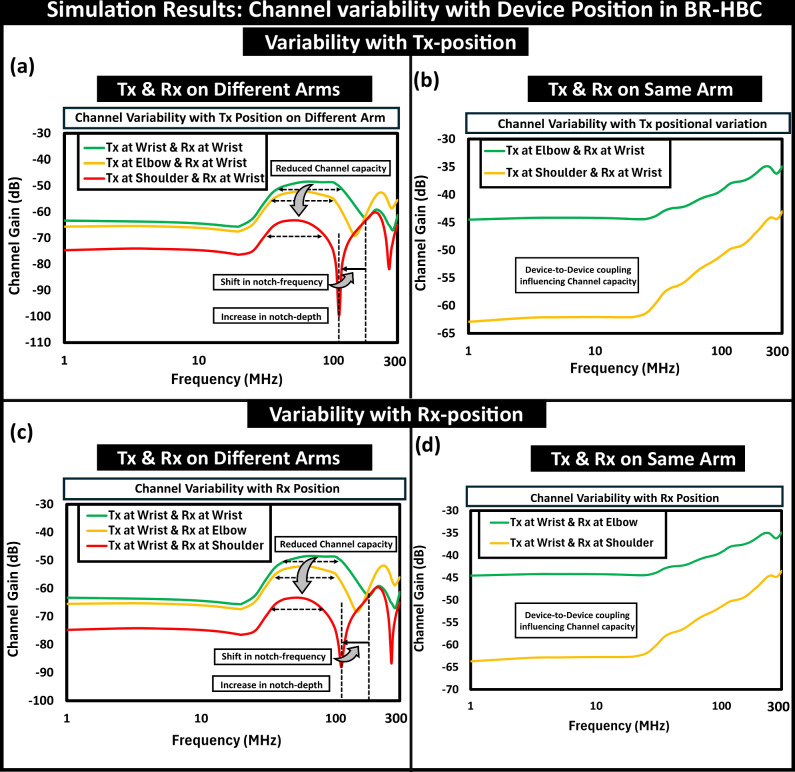
Device position influencing variability in channel capacity. Tx position variation: (**a**) Tx and Rx on different arms, (**b**) Tx and Rx on the same arm. While in the opposite arms, the Tx movement towards the subject’s torso reduces the channel capacity by attenuating the channel gain and operational bandwidth resulting from the combined effect of shadowing of field lines and notch movement in the channel characteristic to a lower frequency. Rx position variation: (**c**) Rx movement on the different arm, (**d**) Rx movement on the same arm. Similar attributes in the channel variability that are observable with Tx movement along arms are noticeable with the positional change of Rx.

The on-body positioning of the communicating devices, i.e., the location of the signal feed (Tx position) and pickup (Rx position) from the surface of the human body, alters the formed EM-wave patterns and thereby leads to variability in channel characteristics, portrayed in Fig. 8. While keeping the Rx fixed at the wrist, the positional change of Tx along the opposite arm towards the shoulder reduces the channel capacity as it reduces the channel gain and operational bandwidth (i.e., the location of the first notch starts moving to a lower frequency), presented in Fig. 8a. The channel gain attenuation happens as the field lines emanating from the Rx-ground get blocked from a conducting surface like the subject’s body, i.e., an equivalent effect, conceptualized and termed body shadowing in EQS^30^. Furthermore, the location of the notches from the first resonance moves to a lower frequency due to the higher relative permittivity of the body tissue (i.e., *ϵ*_*tissue*_  >>  *ϵ*_*air*_). The shift in the Tx position towards Rx in the same arm shows an improved channel gain owing to its behavior dominated by the device-to-device coupling, shown in Fig. 8b. The movement of the Rx towards the torso also exhibits similar trends, captured in Fig. 8c, d.

**Fig. 9:**
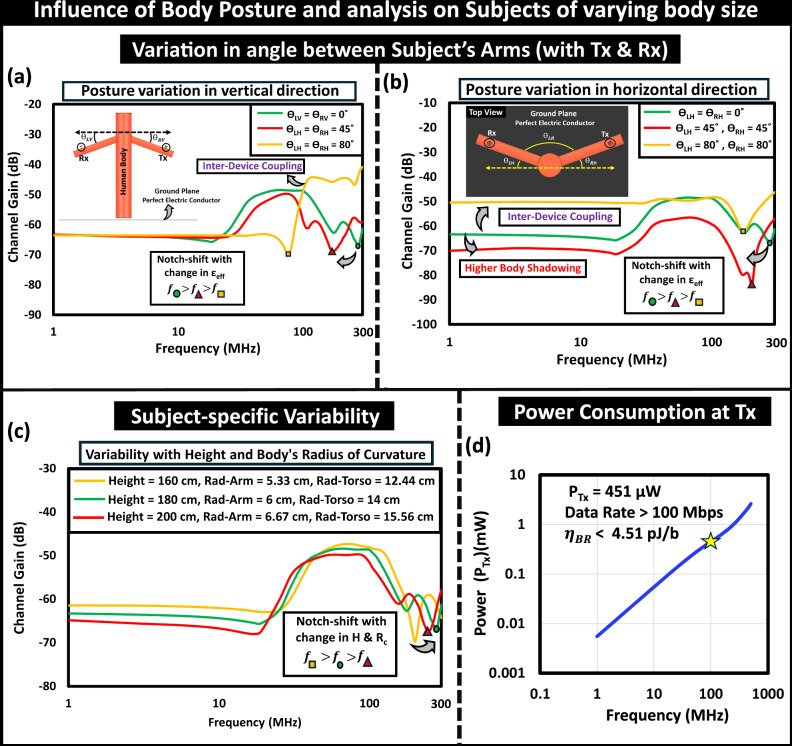
Subject’s body posture influencing channel variability. Variation in angle between subject’s arms: (**a**) Arms rotation in vertical direction, (**b**) Arms rotation in horizontal direction. While keeping the devices in the opposite arms, the arm movement towards the subject’s torso results in a notch shift in the channel characteristic to a lower frequency. **c** Effect of change in subject’s height and radius of curvature of arms and torso, **d** Variation in consumed power at the transmitter highlighting the energy-efficiency of the high-speed communication link.

In addition to device positioning, changes in the subject’s body posture also affect the variability of the channel characteristics. When the on-body locations of the transmitter (Tx) and receiver (Rx) are fixed, the movement of the arms to simulate different postures alters channel behavior, as illustrated in Fig. 9. For example, if the subject rotates arms vertically from a T-pose, the devices move closer to the torso, resulting in reduced channel gain and a shift in the notch frequency to a lower value, conversely, when the arms are held very close to the torso (i.e., *θ*_*LV*_ = *θ*_*RV*_ = 80°), the inter-device coupling becomes more dominant, improving channel gain, as shown in Fig. 9a. A similar trend in notch movement occurs with variations in horizontal posture, as presented in Fig. 9b. The position of the notch depends on the effective permittivity (*ϵ*_*eff*_) encountered along the signal path, which also relies on the line of sight (LoS) between the communicating devices (Tx *&* Rx). Therefore, this relationship varies with changes in body posture. The effect of variation in the subject’s body dimension on channel capacity is captured while keeping the aspect ratio of the human body model consistent, depicted in Fig. 9c. With the increase in subject height in proportion to the radius of curvature of arms and torso, a decrease in channel gain in EQS and a shift in the notch to a lower frequency is observed. While analyzing the change in power consumption at the transmitter with the operating frequency increasing from EQS to BR regime and beyond, presented in Fig. 9d, the viability of energy-efficient (*η* < 4.5 pJ/bit, this theoretical bound of energy efficiency is expected to vary with additional hardware overheads from clock generation, amplifier, mixer etc.) communication at 100’s of Mbps via BR HBC is captured. Besides, the increasing trend of the consumed power confirms the increasing radiative nature of the energy flux from the transmitter at an operating frequency beyond the BR frequency regime.

#### Channel capacity estimation

A detailed breakdown of the procedure for channel capacity calculation is provided: We first determine the frequency at which the frequency response of the BR HBC channel gain (i.e., $G=20\cdot {\log }_{10}\frac{{V}_{Rx}}{{V}_{Tx}}$) reaches its maximum (peak) value. Next, we identify the range of frequencies where the channel gain is reduced by 3 dB from this peak value, as determined from the frequency response plot. This value can also be obtained analytically from the expression of the channel transfer function (*G*(*s*)). Specifically, the 3 dB points can be calculated by locating the frequencies where the magnitude of the transfer function ($G(j\omega )=\frac{{V}_{Rx}(j\omega )}{{V}_{Tx}(j\omega )}$ where *s* = *jω* during steady state under a sinusoidal input excitation), gets reduced to $1/\sqrt{2}$ (or 70.7%) times of its maximum value (*G*_0_(*jω*)) i.e., $G(j\omega )=\frac{{G}_{0}(j\omega )}{\sqrt{2}}$. Finally, the bandwidth (*B*) is calculated as the difference between the lower (*f*_*l*_) and upper (*f*_*h*_) 3 dB points of the frequency response (i.e., the upper and lower cutoff frequencies), expressed as

2 $${{{\rm{BW}}}}={f}_{h}-{f}_{l}$$

After the determination of channel bandwidth, we conducted analysis for the signal-to-noise ratio (SNR), Shannon’s channel capacity (C), bit-error-rate (BER). From the noise sensitivity (*P*_*N*_) of the spectrum analyzer with buffer as input, the amplitude of noise voltage can be obtained as

3 $${V}_{n}=\sqrt{\frac{kT}{{C}_{Leff}}}$$

From the obtained noise voltage, the SNR can be calculated as

4 $${{{\rm{SNR}}}}({{{\rm{dB}}}})=10\cdot {\log }_{10}\left(\frac{{V}_{Rx}^{2}}{{V}_{n}^{2}\cdot 1{0}^{\frac{{{{{\rm{NF}}}}}_{{{{\rm{Rx}}}}}}{10}}}\right)$$

And subsequently, Shannon’s channel capacity is estimated as follows:

5 $${{{\rm{C}}}}={{{\rm{BW}}}}\cdot {\log }_{2}(1+{{{\rm{SNR}}}})$$

#### User’s safety

**Fig. 10:**
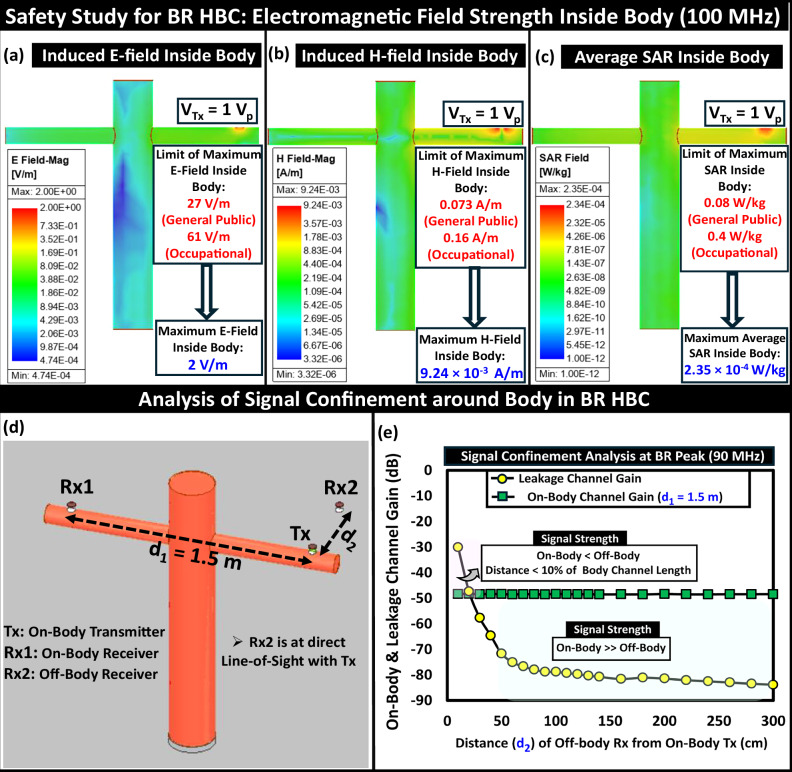
Analyzing safety aspects and leakage profile of BR HBC. **a** Induced E-Field remains an order of magnitude lower than the specified safety thresholds from ICNIRP. **b** Induced H-Field stays several orders below the safety limits. **c** Average Specific Absorption Rate (SAR) lying below the upper threshold. **d** Simulation setup for evaluation of leakage. **e** Comparison of the leakage signal to the on-body signal.

The increase in the consumed transmitter current in the BR frequency regime over EQS demands an analysis of the safety aspects of deploying this technology ubiquitously. In a couple of previous studies^29^, the safety aspects of individuals using EQS-HBC and the recommended upper thresholds for human exposure to radio frequency (RF) electromagnetic fields are discussed as per the guidelines issued by the Institute of Electrical and Electronics Engineers (IEEE)^33^ and the International Commission on Non-Ionizing Radiation Protection (ICNIRP)^32^. With its peak in channel transfer characteristic appearing between 50 to 150 MHz for subjects of height ranging from 160 to 190 cm, for the BR HBC, the upper thresholds are considered to be the induced field and specific absorption limits specified by ICNIRP. We take a numerical simulation-based approach to estimate the induced field strength (electric and magnetic) inside the human body and the Whole-Body Average Specific Absorption Rate (SAR), presented in Fig. 10. We performed electromagnetic analysis on the cross-cylindrical human model in HFSS. The estimated value of the induced E-field inside the body being 13.85X lower (for general public) and 30.5X lower (for occupational) than the maximum allowed E-field (in 10a), the induced H-Field being 7.9X lower (for general public) and 17.31X lower (for occupational) than the maximum H-field limit (in 10b), and the whole-body average SAR turning out to be 340X lower (for general public) and 1702X lower (for occupational) than the specified upper limit (in 10c) at an input voltage of amplitude 1 V corroborates the operational safety of BR HBC.

#### Signal Confinement Analysis

In this section, we investigate the signal confinement behavior of the proposed communication link. The leakage profile around the subject’s body for the proposed communication link during the line-of-sight scenario between on-body Tx and off-body Rx is shown in Fig. 10d, e. It is observed that at the BR peak (90 MHz), the signal strength received off the body (V_*OffBody*_) remains comparable to or higher than the on-body signal strength (V_*OnBody*_) when the distance between the on-body Tx and the off-body Rx becomes less than 15-20% of the fixed on-body channel length spanning 1.5 m. Its $\frac{{V}_{OffBody}}{{V}_{OnBody}}$ being  < 0.1 for an off-body distance of 0.5 m makes the signal confinement of BR HBC comparatively better than traditional radiative wireless communication techniques, where the leakage signal mostly remains comparable to or higher than the received signal strength on the body.

### Experimental results

To verify the trends observed in numerical simulations, we performed measurements on human subjects using wearable devices to demonstrate BR HBC. We use a coaxial SMA connector to couple the signal from the wearable transmitter to the wristband coupler. The coupler’s surface, remaining in contact with the skin, is coated with double-sided conductive copper foil tape in a rectangular shape to emulate the signal patch. Subsequently, we picked up the signal at the receiving end by utilizing another coaxial SMA connector and a similarly designed wristband coupler to the wearable receiver. It is important to note that the signal couplers are not impedance matched to the human body’s input impedance and, hence reflections observed in experiments may differ from the numerical simulation results at higher frequencies. However, even without matching, we can demonstrate that the human body is a high-capacity communication channel for high-speed body-centric communications. We conducted various experiments under different scenarios. The experimental datasets that are plotted for the nominal and repeatable scenarios with statistical conformity provided in terms by plotting the data with mean (*μ*) values and showing the standard deviation (*σ*) with error bars. The upper and lower limits of the error bars are calculated by determining the standard deviation from the mean of five sets of measured data for each data point.

**Fig. 11:**
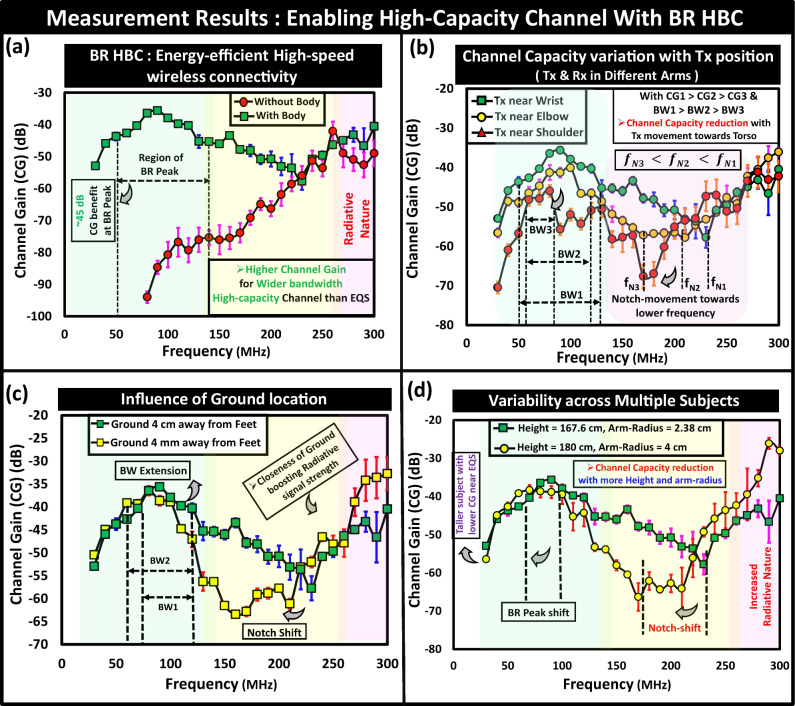
Experimental results. High capacity body-centric communication channel and its variability in Body-Resonance (BR) frequency regime: (**a**) BR Human Body Communication (HBC) offering higher channel gain for wider bandwidth to enable higher channel capacity, (**b**) reduction in body channel capacity with change in the transmitter (Tx) position along subject’s arm towards torso while keeping the receiver (Rx) fixed at the wrist of the other arm, (**c**) influence of location of Ground depicting variation in operational bandwidth (BW), (**d**) influence of subject’s body dimension on channel behavior through study on multiple subjects.

The experimental verification, depicted in Fig. 11a, demonstrates the feasibility of utilizing the human body as a high-capacity wireless channel in the BR regime and highlights its superior performance compared to EQS. This finding supports the theoretical understanding based on field theory and confirms the channel behavior shown in Fig. 5a through numerical simulations. Notably, the presence of the human body provides  ~45 dB benefit in channel gain around the BR peak when compared to over-the-air device-to-device communication scenarios. It is important to note that variabilities in measured datasets are expected to rise at higher frequencies due to increased reflections, as indicated by the rise in *σ*, representing the error bars.

From the circuit theory perspective, the channel gain benefit compared to device-to-device communication, arises from the lower electrical impedance (*Z*_*Body*_) in the forward signal path provided by the Body. Additionally, the broader bandwidth observed in the BR regime over EQS can be explained by its electrically distributed resonance behavior of a lossy transmission line, contributing to a lower Q factor.

The changes in transmitter location on the body while keeping the receiver fixed at the subject’s wrist are shown in the experimental results in Fig. 11b While moving the transmitter towards the subject’s torso in the opposite arm to that of the arm having the receiver, a decrease in body channel capacity in the BR frequency regime is observed. These trends align with the full-wave simulation results, depicted previously in Fig. 8a. A rise in variability within measured datasets (i.e., an increase in *σ*) can happen when the transmitter is positioned very close to the shoulder due to heightened reflections from the subject’s torso at higher operating frequency.

In light of electrical circuit, this channel capacity reduction stems from the decrease in the impedance between Tx-Ground-user’s body (*Z*_*GBTx*_) and an increase in the return path impedance of the Tx-Ground (*Z*_*retTx*_) i.e., ${V}_{Rx}\propto \frac{{Z}_{GBTx}}{{Z}_{retTx}}$ leading to a SNR degradation and bandwidth compression from the shift in notch frequency (i.e., *f*_*N*3_ < *f*_*N*2_ < *f*_*N*1_ where *f*_*N*_ ∝ 1/*C*_*GBTx*_) from the reduced *Z*_*GBTx*_. The detailed derivation of the channel transfer function is provided in the Supplementary discussion 7^28^.

The dependency of body channel capacity on the ground location is portrayed in Fig. 11c which is consistent with the results depicted in Fig. 6d as the closeness of ground boosts the high-frequency radiation. Moreover, user’s proximity to the ground can increase the variability to some extent within measured datasets from the increased radiative nature.

An alternative explanation from the circuit viewpoint resides in the notch shift from the ground proximity to a lower frequency from the increased capacitance *C*_*Body*_ between the body and earth’s ground, leading to a reduced impedance *Z*_*BG*_. Moreover, this *Z*_*BG*_ reduction leads to a low-frequency shift of the boundary between quasistatic to intermediate field regime, and radiative nature appears early.

#### Channel variability across subjects

The measurements of channel gain in the BR frequency regime were conducted on various subjects over a long period. The results shown in Fig. 11d illustrate the variation in channel characteristics across subjects with heights ranging from 165 cm to 180 cm and weights between 60 kg to 80 kg. With the increase in the height and arm-radius of the subject, the channel gain goes down, and the location of the body-resonance peak shifts towards lower frequencies. Furthermore, a noticeable shift in the notch-frequency is observed with increase in arm-radius which remains concurrent with the simulation results depicted in Fig. 9c. Moreover, a taller subject with more arm-radius while acting as a bigger ground to the devices boosts the high-frequency radiation characteristics.

These insights can also be addressed from a circuit viewpoint as the taller subject with higher arm-radius offers higher *C*_*Body*_ resulting in an attenuated received signal. Furthermore, the boundary between EQS and BR encounters a low frequency shift from the increased *l*_*eff*−*Body*_ of the resonator.

Further experimental investigations were conducted to assess the performance of BR HBC for different postures of the subject and the leakage profile around the subject’s body was studied, as detailed in Supplementary Discussion 3^28^. To evaluate the robustness of the proposed body-centric communication link, we characterized its performance in the presence of metallic objects that were in contact with or nearby the user’s body, as illustrated in Supplementary Discussion 4^28^. Additionally, we analyzed the scenario involving multiple users in a BR HBC-enabled Wireless Body Area Network, as depicted in Supplementary Discussion 5^28^.

#### Comparison with traditional wireless

**Fig. 12:**
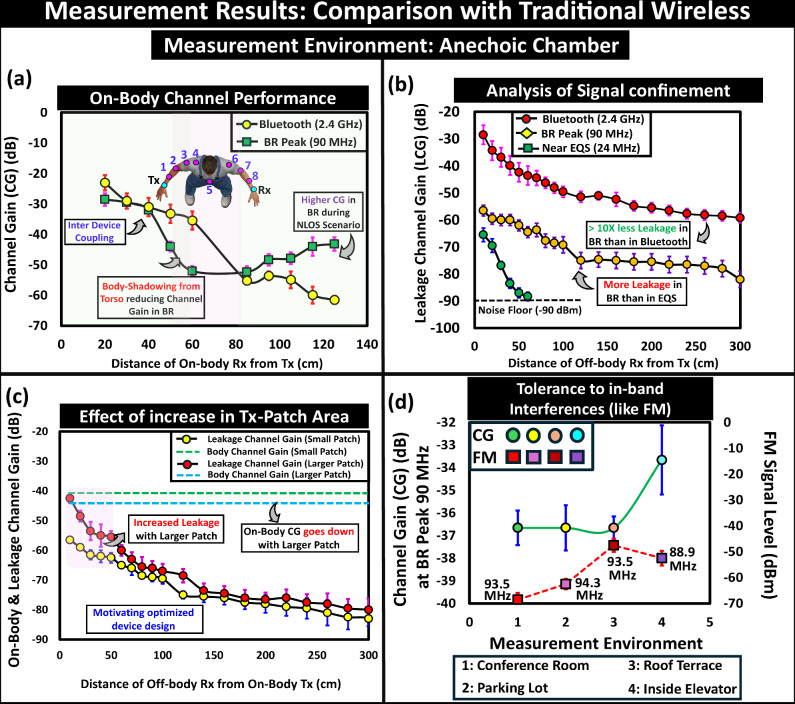
Experimental results. Comparative study of proposed Body-Resonance (BR) Human Body Communication (HBC) with traditional antenna-based wireless communication inside Anechoic Chamber: (**a**) on-body channel performance with variation in on-body Rx position: Better channel gain (CG) with BR HBC during Non-Line-Of-Sight (NLOS) scenarios, (**b**) Analysis of signal confinement between Bluetooth, BR and near Electro-quasistatic (EQS), (**c**) Effect of increase in area of the patch at the transmitter (Tx) on the on-body signal and leakage performance, **d**. Tolerance to in-band interference like Frequency Modulated (FM) signals in different environments.

We compared BR-HBC mode with traditional wireless communication like Bluetooth through two experiments: one studying on-body channel performance and the other investigating off-body leakage characteristics inside anechoic chamber environment. For the conventional wireless communication experiments, we used Bluetooth antennas connected to a handheld RF signal generator as the radio transmitter and a tinySA Ultra spectrum analyzer as the radio receiver in an anechoic chamber environment. During the on-body channel measurements, the test subject maintained a T-pose, holding the transmitter in one arm. The on-body receiver’s position varied from the wrist of the other arm to the near wrist of the arm where the transmitter was positioned, presented in Fig. 15a. The on-body channel gain showed distance-dependent attenuation characteristics away from the transmitter and sharper attenuation during non-line-of-sight scenarios between the transmitter and the receiver, illustrated in Fig. 12a. With the BR HBC, although the on-body channel gain experiences higher attenuation from the body shadowing near the subject’s torso (i.e., reduced *Z*_*GBRx*_ from higher *C*_*GBRx*_ and increased *Z*_*retRx*_ from lower *C*_*retRx*_), it shows better gain over antenna-based radiative communication during non-line-of-sight scenarios.

Off-body leakage analysis was conducted in a line-of-sight scenario between the on-body transmitter and the off-body receiver (placed on a wooden stool setup), shown in Fig. 15b. With the radiative nature of Bluetooth, the leakage channel gain remained at a level of approximately −50 dB over 3 meters from the transmitter, as shown in Fig. 12b. The off-body leakage signal measurement for BR HBC was performed by keeping the RF-spectrum analyzer on a similar stool setup, shown in Fig. 15. The proposed BR-HBC showed faster attenuation of the off-body leakage characteristics away from the transmitter, and its signal level was approximately 20 dB (i.e.,  ~10X reduction in received voltage) lower than the on-body signal strength, increases its communication specificity and making it a energy-efficient modality over radiative transmission, illustrated in Fig. 12b. From circuit perspective, this benefit relies on the communication specificity factor (*CS*) defined as the ratio of on-body to off-body signal strength i.e, *CS*_*EQS*_ > *CS*_*BR*_ > > *CS*_*RF*_. A comprehensive discussion on the quantification of CS benefit of BR HBC over RF-based wireless is provided in the Supplementary discussion 9^28^.

Furthermore, while studying the influence of the coupler area on the relative strength of on and off-body signals, using a larger patch that follows the contour of the subject’s arm, a sharper rise in the off-body channel gain was observed at the expense of slight reduction on-body signal strength (resulting from the increase in effective overlapping area between the signal and ground of the device), depicted in Fig. 12 (c). Another interpretation from the conceptual equivalent circuit suggests that a larger coupler area enhances the effective length (*l*_*eff*_) of the Tx signal electrode, which in turn reduces the impedance between the Tx-signal-to-ground (*Z*_*ppTx*_) and causes a decrease in *V*_*Rx*_ (i.e., *V*_*Rx*_ ∝ *Z*_*ppTx*_), while also resulting in increased signal loss from radiation. This opens up the scope for optimizing the transmitter design, leading to the emergence of optimized form factor devices.

#### Tolerance to Interference

With its electrically conducting nature, in the BR frequency regime, when the human body starts to resonate, it picks up the electromagnetic signals from the interference sources that are present in the surroundings. When the power level of these interferences exceeds the power of the operating signal (i.e., a lower value of Signal-to-Interference Ratio (SIR)), it may cause unreliable reception of digital data packets with a higher bit error rate. With its peak frequency residing in the range of 50 MHz to 150 MHz, the data transmission at the peak of the Body-Resonance can be susceptible to environmental interference in this frequency range, especially from the presence of an FM radio band that ranges from 87.9 to 107.9 MHz in the United States and is divided into 101 channels of 0.2 MHz wide. The variation in the level of measured FM signal level on the subject’s body in comparison to the channel gain at the peak of BR-HBC under different scenarios (indoor and outdoor) are captured in Fig. 12d. The increase in the FM signal level at the outdoor scenarios (especially in case 3: Roof Terrace) i.e., reduced SIR, highlights the need for incorporating techniques towards mitigating interferences. Hence, to fully utilize the higher channel capacity of the BR HBC, previously proposed techniques for interference tolerance like Adaptive Frequency Hopping (AFH)^34^, direct sequence spread spectrum (DSSS), Code-Division Multiple Access (CDMA), and Integrating DDR Receiver as Notch Filter^35^ for strong interference suppression can be used in conjunction to handle in-band interference like FM. The rationale for using BR HBC stems from the need for energy-efficient, high-speed connectivity among battery-powered wearable devices on the body, especially compared to high-frequency wireless systems in the sub-THz and mm-wave ranges. This is discussed in Supplementary Discussion 6^28^, while regulatory compliance as per the FCC’s specified standards for BR HBC is covered in Supplementary Discussion 10^28^.

## Discussion

Body-Resonance HBC offers energy-efficient faster connectivity among miniaturized battery-powered body-connected devices. Towards building up our understanding of complex electromagnetic wave propagation around the human body, we started our analysis with a single-cylinder model emulating human torso and limb. Subsequently, we conceptualize the body channel as a transmission line confirming it through numerical simulation on a complete human model and experimental results on human subjects, covering the maximum channel length (~2 m) for on-body communication. Instead of optimizing the power gain, which requires relevant frequency-dependent matching at the transmitter and the receiver ends, we focused on demonstrating the viability of utilizing the human body as a high capacity channel to support  ~100s of Mbps body-centric communication. With the high impedance capacitive termination at the receiver, we showed the feasibility of wideband communication, spanning from a few tens of kHz to hundreds of MHz.

### Implementation challenges and future scope

To successfully deploy this technology in real-world scenarios, the implementation challenges must be considered. Potential remedies to address these constraints are proposed, serving as a roadmap for future research.

- Susceptibility to Ground Location: Due to variations caused by the proximity of earth-ground-connected or metallic objects in the environment, the communication channel capacity for users of BR HBC can fluctuate. Therefore, a transceiver system capable of adaptively selecting the optimal carrier frequency and power allocation in the BR regime can effectively address this issue.
- Leakage Minimization: Although less radiative than RF, as the carrier frequency increases and the electromagnetic resonance of the human body becomes more pronounced, off-body leakage signals are amplified than in the non-radiative EQS regime. This can lead to reduced specificity and potential security risks to users’ data compared to EQS. To mitigate leakage from the transmitter-body interface, implementing guard rings or traces around sensitive areas can help reduce external interference and leakage currents. This approach, combined with efforts to maximize coupling efficiency (i.e., via matching impedance), will enhance the on-body signal-to-leakage ratio, ensuring greater specificity.
- Non-uniform body potential: With operating wavelength (*λ*) being in the same order of magnitude as Body Dimension, the subject’s body potential may experience variability with distance away from the transmitter, which was otherwise constant in EQS. This can be circumvented by designing location-aware adaptive transceivers in a multi-device wireless network.
- Suited modulation scheme: Selecting an appropriate modulation scheme for a body communication transceiver requires consideration of factors like data rate requirements, power constraints, transmission range, Bit Error Rate (BER), and channel characteristics. Higher data rates require more complex modulation schemes. Moreover, modulation schemes must be robust to channel impairments, such as attenuation and noise. The implementation complexity of the modulator and demodulator circuits is also crucial in deciding the modulation scheme. Hence, a spectrally efficient modulation scheme with higher sensitivity to achieve a low enough BER (<10^−5^) that is energy-efficient and can support higher data rates with reduced interference in adjacent channels, with the capability of handling in-band phase variation, can be a solution towards optimization.
- Handling in-band interferences: Besides the presence of FM interferences, the interference from a digital television (TV) operating in the frequency range from 54–72 MHz; 76–88 MHz, 174–216 MHz is expected in this band; hence, interference detection and dynamic adaptation of parameters like carrier frequency and data rate remain crucial for implementing BR HBC transceivers. Secondly, spread spectrum techniques spread the desired transmitted information across the entire bandwidth and then spread it out at the receiver, which causes the narrowband interference at the receiver to spread out. In contrast, the energy of the signal is spread back. A reduction in channel capacity is expected due to interference; however, even in the presence of interference with the aforementioned techniques and a higher SIR, the overall throughput remains higher than that of EQS HBC.
- Optimizing Communication Energy Efficiency: Designing intelligent transceivers with enhanced sensitivity is crucial for meeting the growing demand for low-power, high-speed wireless communication in battery-operated devices. This necessitates the development of energy-efficient designs for both transmitter and receiver front ends, striving for improvements that can surpass current communication energy efficiencies-  ~sub-10 pJ/bit for EQS HBC and  ~100 pJ/bit for BLE. This foundational physics-based study could also lay the groundwork for the creation of customized, application-specific integrated circuits (ASICs) to effectively integrate this technology into consumer electronic products.

Considering practical use cases from the user’s convenience perspective, we performed measurements with ESD wristbands, having small rectangular metal contacts (2.5 cm  × 3 cm) coated with copper tape as signal couplers. Experimentally verifying the influence of the increased contact area of the signal patch of Tx, presented in Fig. 15c, we concluded that by keeping the polarization pattern at the electrode similar, a larger patch does not give appreciable improvement in on-body channel capacity but increases the off-body leakage at reduced distance between the on-body Tx and off-body Rx. The design flexibility of the Tx patch, not being necessarily required to follow the full contour over the body (that happens in most practical scenarios) enhances its scope of applicability and motivates the design of devices with optimized form factor. Application-specific coupler design for devices located at different parts of the body can facilitate energy-efficient coupling of EM waves to the body surface. Adding tuned impedance matching networks to minimize leakage from the devices is a future goal that can potentially inspire the design of energy-efficient transceivers.

## Conclusion

In this study, we propose Body-Resonance Human Body Communication (BR HBC), a communication method that leverages the electromagnetic resonance phenomena of the human body to establish a low-loss, wide-band, high-speed communication link for miniaturized, battery-powered body area network (BAN) devices. The Body-Resonance frequency range, where the operating signal wavelength approaches the length of the body channel, enables the formation of electromagnetic resonant patterns on the human body and utilizing body channel as a transmission line for high-speed connectivity. The BR HBC exhibits approximately 15–20 dB lower transmission path loss across a wider operational bandwidth, resulting in up to potentially  ~30X improvement in channel capacity compared to EQS. This method offers superior signal confinement (>10 × less leaky) around the user’s body compared to traditional antenna-based wireless radio communication methods, making BR-HBC a promising choice for energy-efficient, high-speed wireless connectivity. We demonstrated the feasibility of enabling a low-loss, wide-band channel for high-speed (~100s of Mbps) body-centric communication. BR HBC has the potential to open up numerous possibilities for diverse body-centric applications that enrich augmented living.

## Methods

This section provides a detailed explanation of the methods used for our numerical simulation and experiments. This will help independent researchers to reproduce our results in the future.

### Setup for FEM-based numerical simulations

**Fig. 13:**
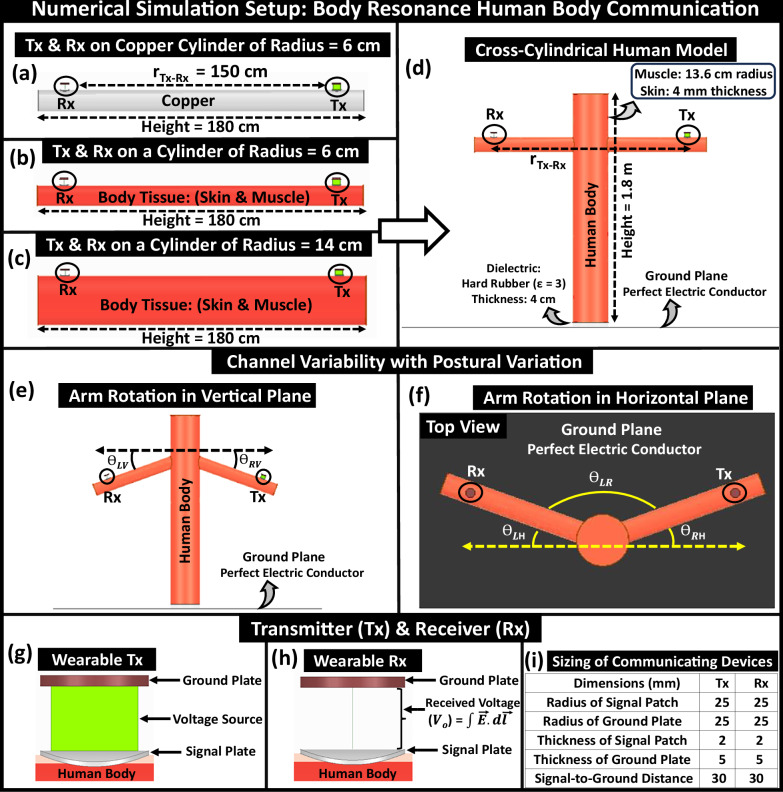
Setup for finite element method (FEM)-based numerical simulation to enable body-resonance human body communication (BR HBC). Simplified Single Cylinder Model with Transmitter (Tx) and Receiver (Rx) on: (**a**) Copper cylinder that replicates human arm of radius 6 cm, (**b**) Body Tissue (skin and muscle) cylinder, (**c**) Tissue cylinder with an increased thickness that emulates human torso of radius 14 cm, (**d**) Cross-cylindrical Full human body model. Subject’s posture influencing channel characteristic: Subject rotates its arms: (**e**) in vertical plane (front view), (**f**) in horizontal plane (top view). Communicating Devices: (**g**) Wearable Transmitter (Tx) and (**h**) Wearable Receiver (Rx) with their signal electrode/patch in contact with skin while respective ground electrodes remain floating. **i** Design specifications of the communicating devices.
- Excitation: A parallel-plate model for the transmitter, presented in Fig. 13g, with the signal electrode connected to the body and the ground electrode, kept floating, is used to excite the human model. Two copper discs with radii of 2.5 cm are used to function as couplers. One of the discs of thickness 2 mm is curved onto the subject’s body to mimic the signal patch, and another disc of thickness 5 mm is used to emulate the ground electrode of a wearable HBC device. The signal and the ground plates are separated at 3 cm. Emulating an AC voltage source of alternating potential difference of amplitude 1 V, in HFSS, a voltage source excitation is placed between the signal and ground plates of the transmitter. Unlike the lumped port, suitable for 50 Ω matched excitation, the assigned voltage source, with its signal and ground plates, constitutes the transmitter (Tx) setup.
- Calculation of Received Voltage: The receiver, illustrated in Fig. 13h, is designed to be structurally similar to that of the transmitter. The potential difference between the signal and the ground plate is calculated by integrating the electric field along a line, joining the two plates of the receiver.
- Calculation of Transmitter Current *&* Power: To measure the current consumption at the transmitter a lumped resistor (R) of 1 Ω is connected in series with the voltage source excitation. Introducing a thin copper cylinder of thickness 0.5 mm and radius 2.5 cm at the midway between Tx-signal and ground, the voltage (*V*_*R*_) across the 1 Ω resistor (i.e., between the middle plate and signal patch) is measured to calculate the root mean square (r.m.s) value of the consumed current (I_*Tx*_ = $\frac{{V}_{R}}{\sqrt{2}}$) at the transmitter. The power consumption at the Tx is calculated as *P*_*Tx*_ = *V*_*Tx*_*I*_*Tx*_ where ${V}_{Tx}=\frac{{V}_{in}}{\sqrt{2}}$, representing r.m.s value of applied input voltage.

- Numerical Simulator *&* Human Body Model: Electromagnetic simulations are performed using ANSYS High-Frequency Structure Simulator (HFSS), which uses Finite Element Method (FEM) to solve Maxwell’s Equations with assigned boundary conditions. A simplified single-cylinder model (shown in Fig. 13a–c) and a cross-cylindrical model of the full human body using two perpendicular cylinders, each of height 180 cm with a radius of 14 cm and 6 cm representing, respectively, the torso and extended arms, is developed, illustrated in Fig. 13d. The use of a simplified model has considerably reduced the computational time and complexity for numerical simulations, whose accuracy was validated previously through a comparison with a detailed model by Maity et al.^29^. The dielectric properties of (i.e., relative permittivity (*ϵ*_*r*_) and conductivity (*σ*)) of the body tissues (skin and muscle) have been taken from the works of Gabriel et al.^36^. The outer shells of the torso and arms are considered to be made up of skin of thickness 4 mm, with the interior muscle emulated by cylinders of radius 13.6 cm and 5.6 cm for the torso and arms. The arms of the cross-cylindrical model have been rotated at different angles to mimic the postural variation of the subject, as presented in Fig. 13e, f. Replicating the scenario of a human standing on the ground, we assumed the human model is 4 cm above a plane with a Perfect E-Boundary assigned to it in HFSS that mimics an infinite ground plane similar to the earth’s ground. With a solution type of modal network analysis and hybrid solver mode, the model is enclosed in a region filled with air and assigned to the Finite Element Boundary Integral (FEBI) boundary condition of the following dimensions: 168 cm  × 292 cm  × 296 cm. Instead of the commonly used radiation boundary which requires a bigger region, the FEBI boundary allows greater flexibility in choosing smaller-sized regions without sacrificing accuracy, leading to faster simulations.

### Experimental setup

**Fig. 14:**
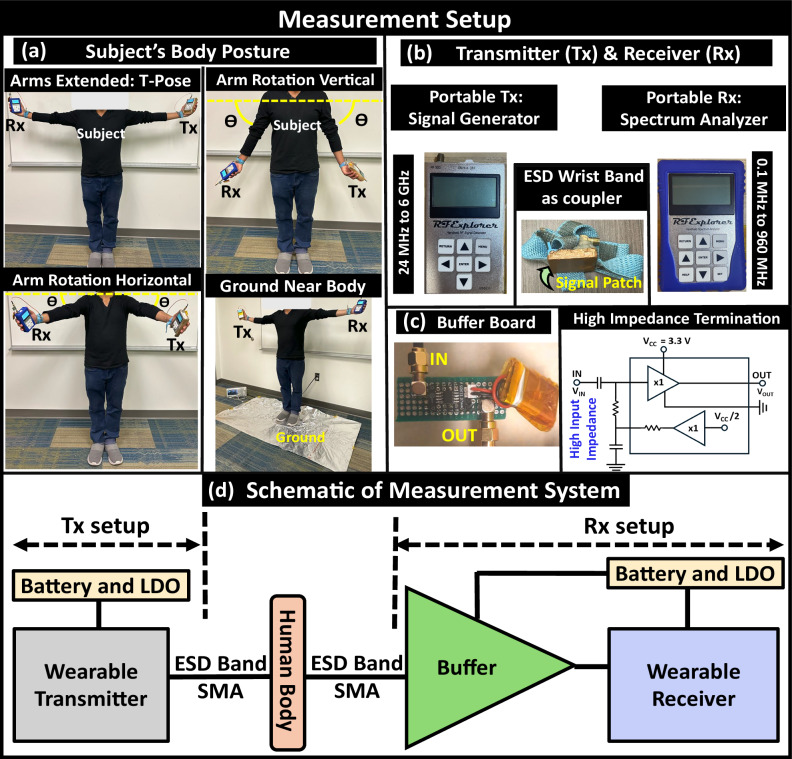
Measurement setup. Subject’s body posture for channel gain measurements: (**a**) the subject holds the portable transmitter (Tx) with its signal coupler in contact with the wrist of one arm, and the portable receiver (Rx) at the wrist of another arm, while emulating a T-pose and various postural variations to capture variability in channel capacity. **b** Portable Signal Generator used as the transmitter. ESD wrist bands used as couplers to couple the signal to and pickup from the subject’s body. Portable Spectrum Analyzer used as the receiver. The receiver setup includes handheld spectrum analyzer together with Buffer. **c** Customized Buffer with high impedance capacitive termination for voltage mode communication. **d** Schematic of the measurement system.

**Fig. 15:**
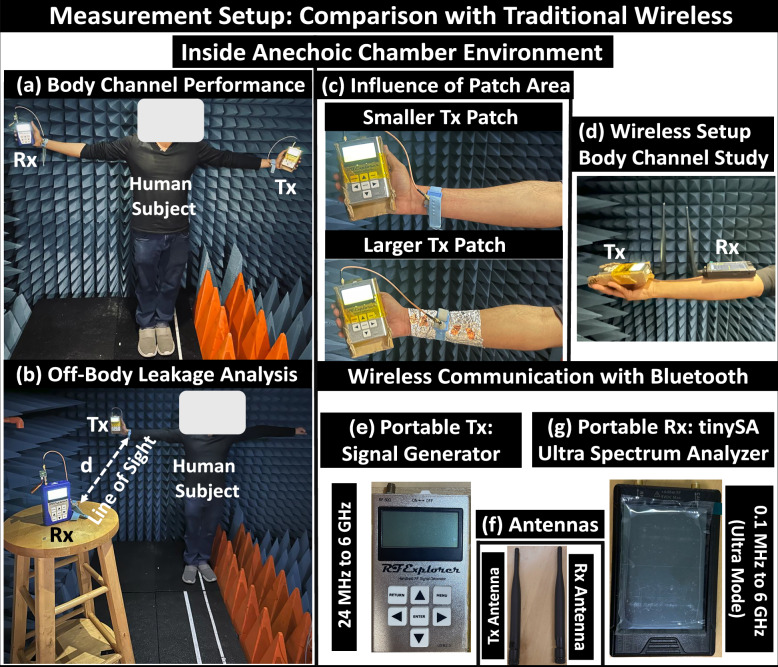
Measurement setup. Comparison of the Body-Resonance Human Body Communication (BR HBC) with traditional wireless inside Anechoic Chamber Environment: (**a**) subject’s body posture during body-channel performance study, (**b**) setup for Off-Body leakage analysis with on-body transmitter (Tx) and Off-body receiver (Rx) (placed at the Line-of-Sight of Tx), (**c**) influence of Tx patch area, (**d**) Setup for wireless communication with Bluetooth, (**e**) Portable signal generator as Tx, (**f**) Tx and Rx antennas, (**g**) TinySA Ultra as portable receiver.

This section portrays the setup used for on-body channel gain and off-body leakage profile measurements. After obtaining informed consent from all participants, we performed experiments involving human subjects while complying with all the guidelines and regulations given by the Purdue Institutional Review Board (IRB Protocol 1610018370). The experiments are performed in two different environments: the first set of experiments, which analyzes the influencing factors towards body channel variability, are executed in a standard conference room, illustrated in Fig. 14 and the second set of measurements is taken in a controlled environment to achieve better noise immunity such as an EM anechoic chamber, presented in Fig. 15. Emulating the scenario of wearable-size communicating devices, battery-operated, handheld transmitter and receiver, shown in Fig. 14b are used. Instead of using a ground-connected Vector Network Analyzer (VNA) that offers an optimistic estimation of channel gain by keeping the grounds of the transmitter and receiver at the same potential, the employed handheld communicating devices closely replicate the realistic channel gain measurements.

- Wearable Signal Transmitter: A handheld RF signal generator (RFE6GEN) from RF explorer, is employed as the signal transmitter that couples the signal to the body. With its operating frequency ranging from 24 MHz to 6 GHz and a resolution bandwidth of 1 kHz, this handheld signal generator (dimension: 11.3 cm  × 7 cm  × 2.5 cm) with its 50 Ω output impedance, is set at a power level of -5 dBm, which ensures that the in-body field and current densities satisfy the ICNIRP safety limits^32^. A thin layer of cut-board casing is taped around the device to avoid any direct contact with the aluminum-made outer casing of the device that is connected to the floating ground of the transmitter.
- Calibration of Wearable Transmitter: With its ground being floated, the RF handheld signal generator requires to be calibrated against a benchtop ground-connected standard. By connecting the transmitter to a Keysight benchtop signal analyzer and fixing the output power of the transmitter and adjusting the frequency from 30 MHz to 300 MHz with a step of 10 MHz, the power shown by the benchtop signal analyzer is recorded and used as the true transmitted power *P*_*Tx*_.
- Signal Coupling at Tx-side: The signal generated by the transmitter is coupled to the user’s body through an ESD wristband linked to the transmitter’s output by a short SMA cable. This cable length is intentionally kept shorter to reduce the radiated field component that can arise, as a longer cable would increase the transmitter’s effective length (*l*_*eff*_), potentially shifting transmitter resonance peaks into the BR frequency range and resulting in higher channel gain than would be observed in realistic wearable-wearable communication conditions.
- Wearable Signal Receiver *&* Buffer: A handheld spectrum analyzer (WSUB1G+) from RF Explorer, having an operating frequency ranging from 100 kHz to 960 MHz and a frequency resolution of 1 kHz, is used as the receiver. Similar isolation measures that were taken previously for the wearable transmitter are adopted for the receiver to prevent the subject from shorting to the receiver ground. This spectrum analyzer, with its 50 *Ω* input impedance, requires a buffer at its input for high-impedance capacitive terminated measurements. A buffer board is customized using BUF602 (a high-speed, wide bandwidth buffer IC from Texas Instruments), presented in Fig. 14c. A small-sized 3.7 V (nominal) rechargeable lipo battery powers up the buffer circuit. With its high bandwidth of 1 GHz and high slew rate of 8000 V/*μ*s, it is suitable to handle high-speed AC signals. The high-impedance capacitive termination is ensured by the input impedance of the buffer, which is about 2 pF capacitive termination at the input side.
- Receiver Setup Calibration: To ensure the accuracy of the experiment results, the errors from the wearable receiver, and the characteristics of the buffer are de-embedded. The calibration processes are described as follows:
- Calibration of Wearable Spectrum Analyzer: The handheld receiver is calibrated by linking it to a Keysight signal generator, a benchtop standard. The output power of the benchtop signal generator is adjusted from -85 dBm to -25 dBm in increments of -5 dBm while varying the frequency from 30 MHz to 300 MHz, and the power difference observed between the two devices is noted. Once the received power is measured during the experiment, software interpolation is conducted to determine the correction factor for the receiver at the specific frequency of interest, referred to as *C*_*Rx*_.
- Calibration of Buffer: The input of the buffer is connected to the output of the signal generator while the output is connected to the benchtop signal analyzer. The difference between the power, shown by the benchtop signal analyzer and the calibrated real transmitted power is recorded as the correction factor for the buffer, denoted as *C*_*Buffer*_.
- Signal pickup at Rx-side: The signal pickup from the intended body locations is performed using another ESD wristband connected to the signal electrode of the receiver setup by a short SMA cable to minimize radiation losses. It is worthwhile noting that with the single-ended pick up the location of receiver’s ground relative to the human body remains decisive as it decides the received signal strength (*V*_*Rx*_) based on the ratio of effective load impedance (*Z*_*Leff*_) to the return path impedance (*Z*_*retRx*_) at the receiver since, ${V}_{Rx}\propto \frac{{Z}_{Leff}}{{Z}_{retRx}}$.
- Devices for measurements with Bluetooth: In experiments with traditional wireless communication methods, such as Bluetooth, we utilize two 2.4 GHz omnidirectional antennas, shown in Fig. 15f. These antennas function within the frequency range of 2.4 - 2.485 GHz and possess linear vertical polarization, a gain of 6 dBi, VSWR less than 2.0, and an impedance of 50 ohms. We have connected one antenna to the RF signal generator to serve as the transmitter setup, while the other antenna is connected to the TinySA Ultra spectrum analyzer, which has a measuring range from 100 kHz to 5.3 GHz in its Ultra mode and is employed as a wearable receiver, illustrated in Fig. 15d–g.

#### Measurement procedure

The performance of the body channel has been experimentally studied in the BR regime by sweeping frequencies for specific positions of the transmitting and receiving devices on the body and by sweeping distances to capture the channel variability. Channel gain measurements in the absence of the human body were conducted by placing the transmitting and receiving devices at a certain distance on two wooden stools. The leakage profile around the subject’s body was measured using a wearable transmitter worn on the body and keeping the handheld receiver on a wooden stool setup. Subsequently, the channel gain for each measurement is calculated from the recorded power (*P*_*Rx*_) shown by the handheld receiver by subtracting the transmitted power (*P*_*Tx*_) from it while taking the correction factors from the calibration of the devices (*C*_*Rx*_) and buffer (*C*_*Buffer*_) into account. The schematic of the measurement system shown in Fig. 14c illustrates the signal processing pipeline.

### Reporting summary

Further information on research design is available in the Nature Portfolio Reporting Summary linked to this article.

## Supplementary information

Supplementary Information

Description of Additional Supplementary Files

Supplementary Movie 1

Supplementary Movie 2

Supplementary Movie 3

Reporting Summary

## Data Availability

The datasets that support the plots of numerical simulation and experimental results along with other findings that are presented in this paper, are available from the corresponding author upon reasonable request. Source datasets are presented in this paper.

## Code availability

Custom codes that are used to process the data are available from the corresponding author upon reasonable request.
