# Supplementary material for: Body-resonance: transmission line-like wireless links enabling high-speed wearable communication
Source: Commun Eng. 2025 Dec 20;5:1. doi: 10.1038/s44172-025-00533-z (PMC12764851; doi:10.1038/s44172-025-00533-z)
Supplement: Supplementary file 1 — Supplementary Information [file 44172_2025_533_MOESM1_ESM.pdf]

Supplementary Information: Body-Resonance: Transmission Line-like Wireless Links Enabling High-speed Wearable Communication

Samyadip Sarkar[1], Qi Huang[1], Sarthak Antal[1], Mayukh Nath[1] and Shreyas Sen[1*]

[1]Elmore Family School of Electrical and Computer Engineering, Purdue University, West Lafayette, 47907, Indiana, USA.

*Corresponding author(s). E-mail(s): shreyas@purdue.edu;
Contributing authors: sarkar46@purdue.edu;
huan2065@purdue.edu; santal@purdue.edu;
nathm@alumni.purdue.edu;

Contents:

- **Supplementary Discussion 1: Current Density and H-field Distribution**
- **Supplementary Discussion 2: Behavioral Difference of BR HBC in Machine-Machine (M2M) vs Wearable-Wearable (W2W) Scenario**
- **Supplementary Discussion 3: Performance Analysis of BR HBC**
- **Supplementary Discussion 4: Subject's proximity to Metallic Object**
- **Supplementary Discussion 5: BR HBC in Multi-Human Handshaking Channel**
- **Supplementary Discussion 6: BR HBC in context of the conventional wireless spectrum**
- **Supplementary Discussion 7: Relating Field-based understanding to Circuit Model**
- **Supplementary Discussion 8: Influence of Tissue Properties on Channel Characteristics**
- **Supplementary Discussion 9: Communication Specificity Comparison of BR HBC with Radiative Wireless**
- **Supplementary Discussion 10: Regulatory Constraints for BR HBC**
- **Supplementary Discussion 11: Comparison of BR HBC with prior related studies**
- **References**

Supplementary Material

Supplementary Discussion 1: Current Density and H-field Distribution:

We present a comparative analysis of the current density and magnetic field distribution via numerical electromagnetic analysis between the Electro-quasistatic (EQS) and Body-Resonance (BR) frequency regimes, illustrated in Supplementary Fig. 1.

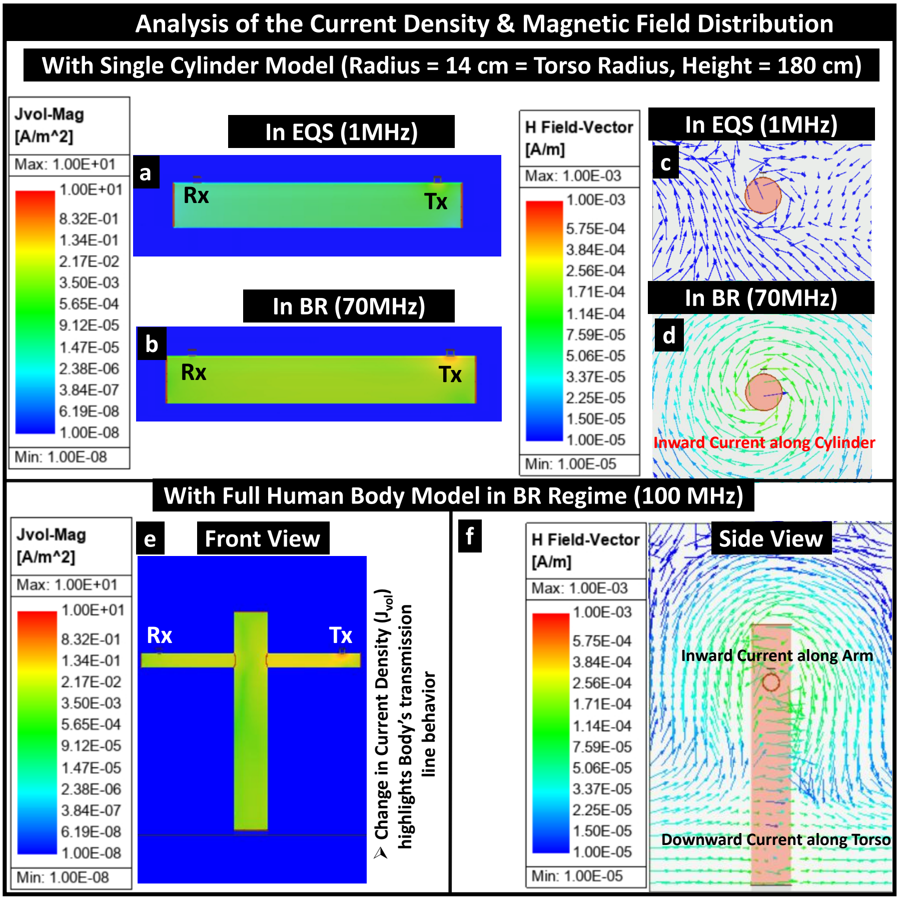

Supplementary Fig. 1 Analysis of Current Density and Magnetic Field Distribution: with Single Cylinder Model: The complex magnitude of the current density (J_{vol}) over the body volume: **a.** In EQS, **b.** In Body-Resonance, Comparison of the magnetic field vector: **c.** In EQS, **d.** In BR, **with Cross-Cylindrical Human Body Model in BR at 100 MHz: e.** Current Density variation over the volume of the body, **f.** H-field vector

When the operating wavelength (λ) greatly exceeds the maximum dimension of on-body communication channels (i.e., $\lambda >> l_{Body}$) in the EQS regime,

a consistent potential exists throughout the cylindrical body model, resulting in a uniform current distribution, shown in Supplementary Fig. 1 (a). Conversely, in the BR regime, where λ is comparable to the body channel length, there is an increased non-uniform current distribution within the conductor. This variability in current density inside the volume of the human body affirms its conceptual model as a lossy transmission, delineated in Supplementary Fig. 1 (b, e).

Furthermore, the direction of the H-field indicates the direction of the current carried by the human body. In the EQS regime, the induced H-field is significantly lower than the induced E-field with an electric dipole, allowing us to disregard the influence of the H-field, shown in Supplementary Fig. 1 (c). Nonetheless, the shift in the direction of the H-field vector, as shown in Supplementary Fig. 1 (d, f), confirms the unbalanced, lossy transmission line nature of the human body, with the body as a signal conductor and the earth's ground acting as a ground conductor. Along with the variation in H-field, the variation in Electric Field & Energy Flux Density around human body demonstrate the transmission line behavior of human body, presented in Supplementary Movie 1 with this study https://github.com/SparcLab/BodyResonanceHBC. Additionally, we provided an animation plot illustrating the variation in H-field with frequency as Supplementary Movie 2.

Supplementary Discussion 2: Behavioral Difference of BR HBC in Machine-Machine (M2M) vs Wearable-Wearable (W2W) Scenario

In the context of capacitive HBC, communication devices are known to be classified based on the size of their ground and their coupling to the earth's ground as follows: (a) Machines: These devices have their ground connected to the earth's ground, resulting in higher channel gain that solely depends on the path loss incurred from the body channel. They can also be referred to as ground-connected devices. (b) Wearables: These small form factor, battery-operated devices experience high impedance in their return path (Z_{retTx}, Z_{retRx}) that results from lower return path capacitance ($C_{retTx}, C_{retRx} \leq 1$ pF)[1] due to reduced parasitic ground coupling and can also be referred as ground-floated devices. (c) Tabletop devices: These devices, although small yet when placed on a table, experience unrealistically lower impedance in the return path in comparison to the wearable-to-wearable scenario owing to their return path capacitance lying in the range of 100 -200 pF[2]. For EQS HBC, with voltage mode communication and capacitive termination at the receiver, the change in the setup from Machine-Machine (M2M), Wearable-Wearable (W2W) to Tabletop results in primarily the change in the received signal level (i.e., $V_{Rx-M2M} >> V_{Rx-Tabletop} >> V_{Rx-W2W}$).

However, in BR HBC, the operational frequency range, i.e., the location of the BR peak, changes with the setup as the M2M/M2W setup shifts the peak to a lower frequency than the W2W setup. With the M2M/M2W setup, the

human body acts like a quarter wave monopole (i.e., $f_r \propto \frac{1}{4l_{Body}}$) in proximity to earth's ground, which may result in reduced channel capacity and peak location around 37.5 MHz for a subject height of ∼2 m. In contrast, in the W2W setup, the increased Z_{retTx} and Z_{retRx} i.e., reduced C_{retTx} and C_{retRx} respectively shift the body resonance peak to a higher frequency and enable the human body to act like an imperfect resonator resembling a lossy transmission line with broadband (i.e., low Q) resonance, resulting in increased channel capacity and peak location around 80 MHz or higher for a subject height of ∼2 m.

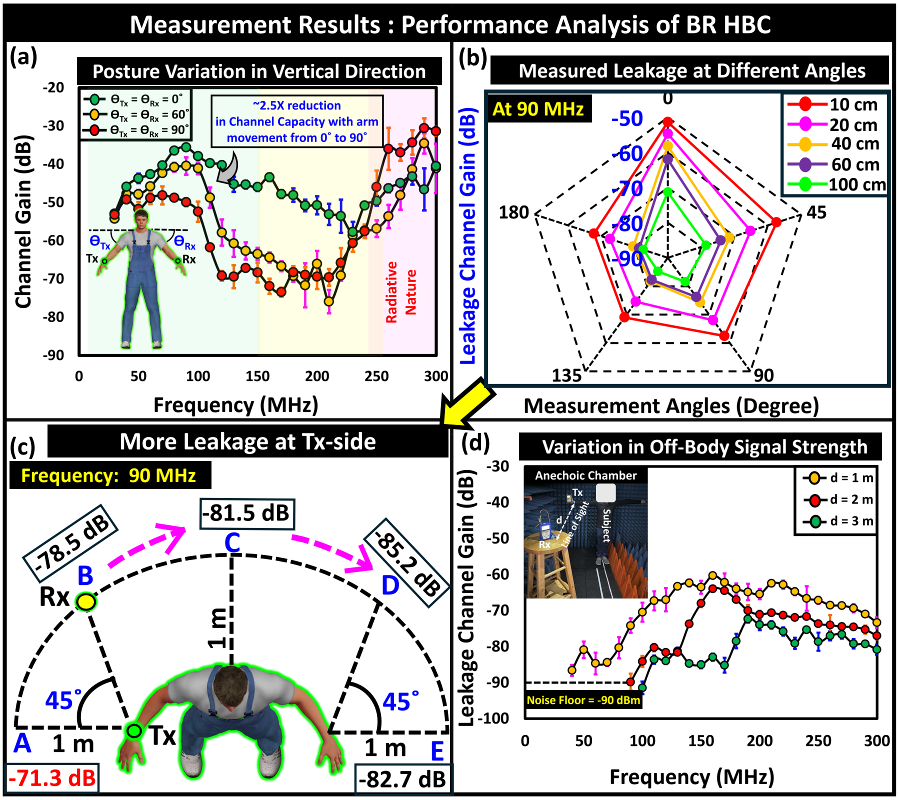

Supplementary Fig. 2 :**Performance Analysis of BR HBC: a.** Channel Capacity variation with change in subject's body posture, **b.** Measured leakage at different angles and at different distances away from the Tx and user's body , **c.** Illustrating more leakage at Tx-side at the BR peak frequency of 90 MHz, **d.** Variation in leakage over different freuqncies in BR regime.

Supplementary Discussion 3: Performance Analysis of BR HBC

To investigate the performance of BR HBC, we characterized the BR HBC channel across different postures, illustrated in Supplementary Fig. 2 (a). Our findings align with previous theoretical insights that are presented in the main manuscript, indicating that the proximity of the Tx and Rx to the subject's torso—specifically, as the arm angle gradually changes from 0° in the T-pose to 90° with the arms relaxed—results in a ∼2.5X reduction in channel capacity. This reduction is attributed to reduced SNR and attenuation in operational bandwidth due to a notch shift to lower frequencies resulting from increased effective permittivity (ϵ_{eff}). Interestingly, despite this reduced channel capacity in the arm-relaxed position, it has the potential to outperform the supported data rate of the EQS HBC under identical conditions. This variabilities can be tackled by designing adaptive transceivers systems that can dynamically select the optimal carrier frequency for power allocation with a suited link margin, which lies beyond the scope of this work and inspires future research. Now, to characterize the signal confinement, we performed leakage measurements around BR peak (90 MHz) at different distances and at different angles around user's body, presented in the form of a radar plot in Supplementary Fig. 2 (b). The results confirm our understanding of increased leakage near the body, with a corresponding attenuation observed at greater distances. Additionally, as shown in Supplementary Fig. 2 (c), approximately 10 dB higher leakage is detected at the transmitter side at a distance of 1 meter in an open area. Furthermore, the leakage profile across various frequencies in the BR regime is captured inside an anechoic chamber, shown in Supplementary Fig. 2 (d).

Supplementary Discussion 4: Subject's proximity to Metallic Object:

In the realm of human-machine interaction[3,4], the practical deployment of this technology necessitates an examination of the robustness of the proposed wireless link. The underlying principle of operation of BR HBC relies on near-intermediate electric field-based coupling, making it sensitive to the presence of metallic objects in contact or proximity to the user's body. Such influences from metal structures can affect the received signal level and operational bandwidth, consequently impacting the channel's capacity for high-speed communication, as illustrated in the conceptual schematic shown in Supplementary Fig. 3 (a). It can be inferred that when a BR HBC user comes into contact with a conducting or metallic object, a degradation in SNR and a reduction in bandwidth are to be expected due to an increase in effective channel length leading to higher loading, as depicted in Supplementary Fig. 3 (b).

Furthermore, the results from numerical simulations and measurements presented in Supplementary Fig. 3 (c) corroborate this understanding, revealing increased fluctuations in channel performance at higher frequencies

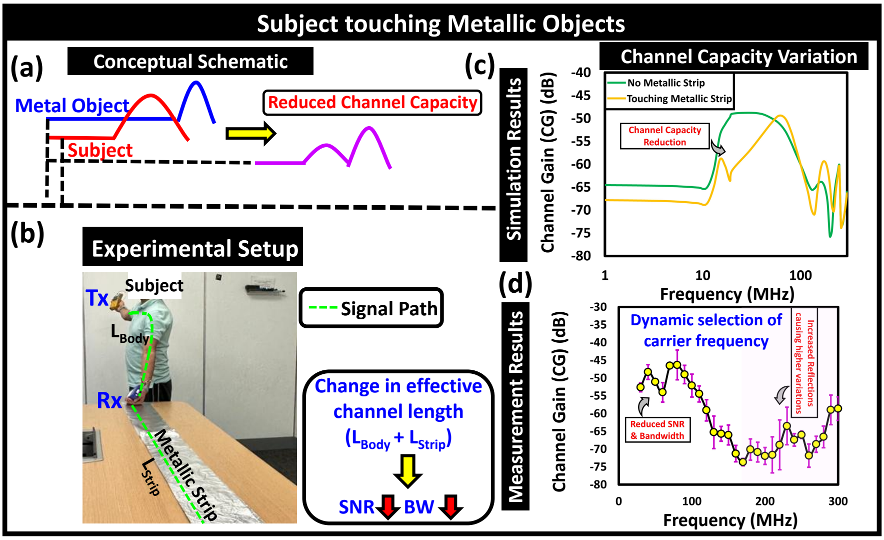

Supplementary Fig. 3 :**Influence of Conducting Objects presence in surroundings: a.** Conceptual Schematic, **b.** Experimental Setup **c.** Simulation Results showing channel capacity reduction as a result of SNR degradation and attenuation in operational bandwidth from the increased effective length of the communication channel. **d.** Measured dataset showing increased variations in higher frequencies owing to the increase reflections from the metallic obejcts. The solution to these variabilities lies in dynamic selection of carrier frequency for optimum power allocation.

resulting from enhanced reflections in the presence of additional conductive materials. These variabilities can be addressed through dynamic carrier frequency selection for optimal power allocation and adaptive matching at the devices. Now, from a circuit perspective, the proximity of conductive structures to the grounds of both the transmitting and receiving devices is expected to improve the received signal strength by lowering the impedance of their return paths (Z_{ret}), as illustrated by the relationship: $V_{Rx} \propto \frac{1}{Z_{ret-Tx} \cdot Z_{ret-Rx}}$. However, as the metallic object approaches the user's body, a significant shift occurs in the frequency boundary that distinguishes the non-radiative Electro-quasistatic (EQS) regime from the increased radiative nature of BR frequency regimes. This shift occurs at a lower frequency due to the increased body capacitance (C_{Body}), which modifies the relationship as follows: $f_{EQS-BR} \propto \frac{1}{C_{Body}}$.

Supplementary Discussion 5: BR HBC in Multi-Human Handshaking Channel:

In wireless communication, investigating the performance of a communication channel when it scales up to multiple users in a body-area network is crucial and hence portrayed in Supplementary Fig. 4. The conceptual schematic of such a channel is presented in Supplementary Fig. 4 (a), where a taller subject

(height = 190 cm) with a transmitter (Tx) operating in the BR regime shakes hand with a shorter subject (height = 168 cm) with a receiver, causes a partial overlapping of BR peaks of the individuals as illustrated in the experimental setup in Supplementary Fig. 4 (b). The channel's measured performance is shown in Supplementary Fig. 2 (c). From the field-theory perspective, for simplicity, assuming the body-channel length being L_{Body} for an individual, the increased no of users (N) in the network results in the increased channel length ($L_{net} = N \cdot L_{Body}$) that leads to low-frequency shift of the boundary between quasistatic near-field ($k{\cdot}r << 1$)-to-reactive electromagnetic intermediate field ($k{\cdot}r > 1$)-to- radiative far field ($k{\cdot}r >> 1$) limits as r increases with increased no. of users $r \propto N \cdot L_{Body}$. Now, from a circuit theory perspective, this scenario can also be viewed as resonators getting cascaded during such interaction and with more no. of network users, the more degradation of signal strength (V_{Rx}) is expected in comparison to a single user due to an increase in the effective body-ground coupling (i.e., Z_{BG}) as $V_{Rx} \propto Z_{BG} \propto \frac{1}{C_{Body}}$. Hence, with an attenuated SNR, the power efficiency may go down, but the throughput remains higher compared to its EQS and RF under identical scenarios.

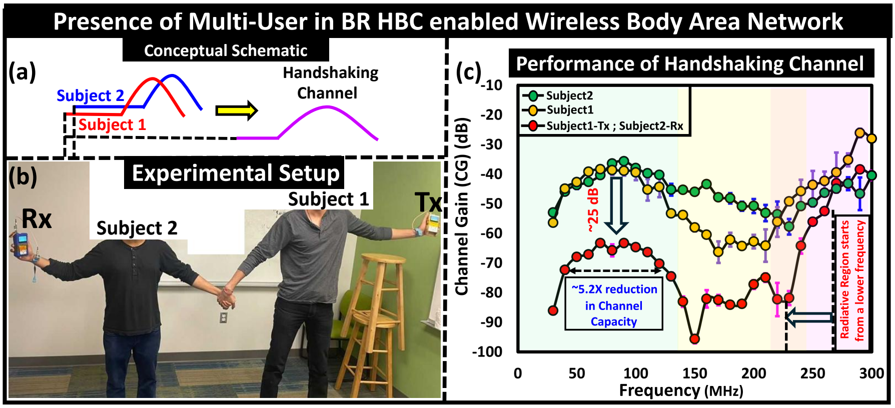

Supplementary Fig. 4 :Presence of Multi-User in a BR HBC Network: a. Conceptual Schematic illustrating the overlap of the BR regime for the two subjects, **b.** Experimental Setup **c.** Performance of BR HBC in a handshaking channel.

Supplementary Discussion 6: BR HBC in context of the conventional wireless spectrum

There has been a significant trend towards the design of high-frequency wireless systems, particularly in the sub-THz and mm-wave ranges. However, these frequencies may not be ideal when considering the need for energy-efficient, high-speed connectivity among battery-powered wearable devices located around the body. They pose challenges such as high transmission path loss, a requirement for line-of-sight, and sensitivity to obstacles, etc. which

can impede communication coverage and reliability. The constraints associated with enabling body communication links at sub-THz and mm-wave bands are depicted below:

1. Higher Transmission Path Loss: mm-wave systems (frequencies between 30-300 GHz) with wavelengths ranging between 1-10 mm and sub-THz systems (frequencies between 300 GHz-3 THz) with wavelengths ranging between 100 µm-1 mm, owing to their orders of magnitude shorter wavelength compared to the human body dimension, incur higher signal attenuation (reduced signal strength) as it travels around the human body.

2. Scattering and Diffraction: High frequencies are also more susceptible to scattering and diffraction, making it difficult to maintain a stable connection in complex environments.

3. Requirement of Line-of-Sight (LOS) : With mm-wave in the higher part of the radio wave spectrum, sub-THz frequencies being even higher in the radio wave spectrum and extending into the terahertz region, these frequencies are more susceptible to blockage and require a clear line of sight between the transmitter and receiver for reliable communication. During the commonly encountered Non-Line-of-Sight scenario the higher loss incurred at these frequencies may not fit the bill with the supported link budget of battery-powered wearable devices.

4. Limited Coverage Area: The higher attenuation during propagation around the human body and LOS requirements for reliable operation results in significantly shorter communication coverage compared to the proposed BR HBC.

5. Higher Signal Leakage: With the operating frequency making transition to the mm-wave and sub-THz band, the increased far-field component of the transmitted signal increases the off-body signal which may pose security threats to the user's personal data.

6. Higher sensitivity around Body & Signal Degradation: The signal transmitted in the sub-THz, mm-wave frequency bands are highly susceptible to their change in transmission path i.e. they get more easily diffracted (bent) around the human body, leading to an increased interference between multiple propagation paths that can lead to signal degradation.

7. Technological Challenges: The limited availability of suitable components such as antennas, transceivers, and amplifiers etc. and need for modulation schemes making it more challenging to design and implement wireless systems for body area network devices at these frequencies.

Supplementary Discussion 7: Relating Field-based understanding to Circuit Model

The fundamental-physics based study being the primary focus of this work, relating the field theory based understanding with the proposed conceptual model remains one of the central focus of this work. With single-ended excitation-pickup and voltage mode signaling, the transfer function for the impedance-based model, presented in Fig. 5, can be formulated as follows:

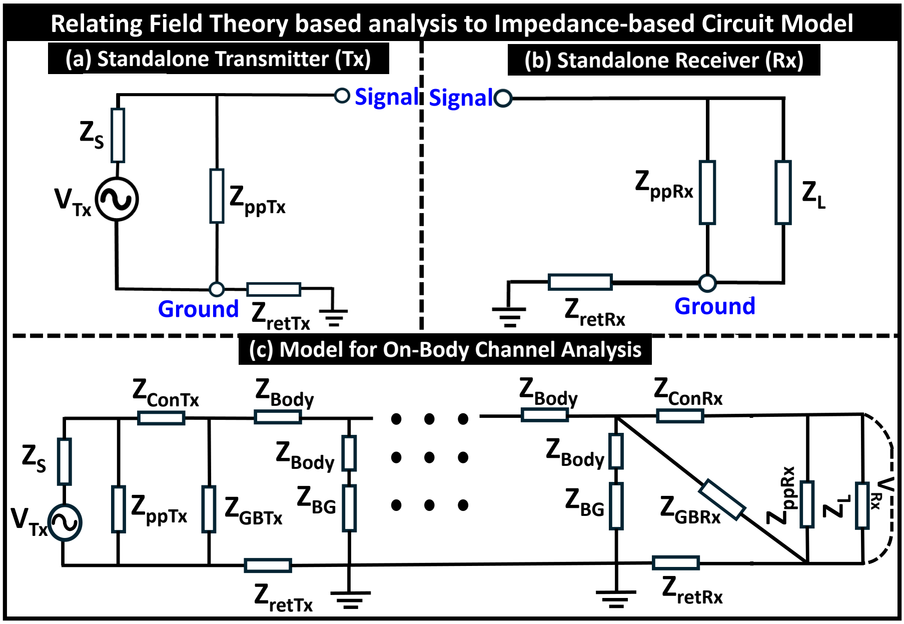

Supplementary Fig. 5 :Conceptual Simplified Impedance-based Circuit Model: at the transmitter (Tx) side: with applied input ac-source being $\mathrm{V_{Tx}}$, Source impedance being $\mathrm{Z_S}$, signal-to-ground electrode impedance being $\mathrm{Z_{ppTx}}$, impedance between the floating ground of the Tx to earth's ground (i.e., return path impedance) being $\mathrm{Z_{retTx}}$, contact impedance between the signal electrode of Tx and user's body being $\mathrm{Z_{ConTx}}$, impedance between floating ground of the Tx to subject's body being $\mathrm{Z_{GBTx}}$; at the receiver (Rx) side: signal-to-ground electrode impedance being $\mathrm{Z_{ppRx}}$, impedance between the floating ground of the Rx to earth's ground (i.e., return path impedance) being $\mathrm{Z_{retRx}}$, load impedance being $\mathrm{Z_L}$, contact impedance between the signal electrode of Rx and user's body being $\mathrm{Z_{ConRx}}$, impedance between floating ground of the Rx to subject's body being $\mathrm{Z_{GBRx}}$, impedance of the body channel being $\mathrm{Z_{Body}}$ conceptualized as a transmission line with RLGC parameters, impedance between body to earth's ground being $\mathrm{Z_{BG}}$: **a.** Model for standalone Tx, **b.** Model for standalone Rx **c.** Model for On-Body Channel Analysis

For an applied input excitation being V_{Tx}, the device coupling efficiency ($\eta_{Tx-Body} = \frac{V_{Body}}{V_{Tx}}$) can be formulated as follows:

$$\eta_{\mathrm{Tx-Body}} = \frac{1}{A \cdot B + D} \tag{1}$$

where, A, B, D can be represented as

$$A = \left(\frac{Z_{\mathrm{GBTx}} Z_{\mathrm{Body(eff.)}} + Z_{\mathrm{ConTx}} \left(Z_{\mathrm{GBTx}} + Z_{\mathrm{Body(eff.)}} \right)}{Z_{\mathrm{GBTx}} Z_{\mathrm{Body(eff.)}}} \right) \tag{2}$$

$$B = \left(\frac{Z_{\mathrm{ppTx}} Z_{\mathrm{ConTx}} + Z_S \left(Z_{\mathrm{ConTx}} + Z_{\mathrm{ppTx}} \right)}{Z_{\mathrm{ppTx}} Z_{\mathrm{ConTx}}} \right) \tag{3}$$

$$D = \left(\frac{Z_S Z_{\mathrm{retTx}}}{Z_{\mathrm{Body(eff.)}} Z_{\mathrm{ppTx}}} - \frac{Z_S}{Z_{\mathrm{ConTx}}} + \frac{Z_{\mathrm{retTx}}}{Z_{\mathrm{Body(eff.)}}} \right) \tag{4}$$

Z_S is the source impedance of the transmitter, $Z_{ConTx}(\omega)$ denotes the contact impedance between the Tx-signal electrode and Body and Z_{ppTx} presents the signal plate-to-ground plate impedance and Z_{GBTx} stands for the parasitic impedance between Tx-ground and body. Hence, $\eta_{Tx-Body}$ can be maximized by reducing Z_S, Z_{ConTx} and by increasing Z_{ppTx}, Z_{GBTx}.

$Z_{Body-L1}$ and $Z_{Body-L2}$ can be conceptualized as a segments of lossy transmission line and formulated as follows

$$Z_{Body}(\omega) = R_{Body}(\omega) + j \cdot X_{Body}(\omega)$$

and Z_{BG} is the parasitic impedance between the user's body to earth's ground. $\mathrm{Z_{Body(eff.)}}$ is defined as: $\mathrm{Z_{Body(eff.)} = Z_{Body-L1} + Z_{Body-L2} + Z_{BG}}$ Now, with voltage mode pickup at the receiver, the output voltage can be represented as follows:

$$V_{Rx}(\omega) = \left(\frac{Z_P}{Z_1}\right) \cdot \left(\frac{Z_1 \parallel Z_{GBRx}}{Z_1 \parallel Z_{GBRx} + Z_{retRx}}\right) \cdot V_{Body} \tag{5}$$

where $Z_P = (Z_{ppRx} \parallel Z_L)$ represents the effective impedance between the signal-to-ground of the Rx; $Z_1 = (Z_{ConRx} + Z_P) = (Z_{ConRx} + Z_{ppRx} \parallel Z_L)$ and Z_{ConRx} denotes contact impedance between the user's body and Rx's signal electrode. At the receiving end the efficiency of voltage pickup ($\eta_{Body-Rx}$) can be expressed as:

$$\begin{aligned}\eta_{Body-Rx} = \frac{V_{Rx}(\omega)}{V_{Body}(\omega)} &= \left(\frac{Z_P}{Z_1}\right) \cdot \left(\frac{Z_1 \parallel Z_{GBRx}}{Z_1 \parallel Z_{GBRx} + Z_{retRx}}\right) \\ &= \left(\frac{Z_{ppRx} \parallel Z_L}{Z_{ConRx} + Z_{ppRx} \parallel Z_L}\right) \cdot \\ &\left(\frac{(Z_{ConRx} + Z_{ppRx} \parallel Z_L) \parallel Z_{GBRx}}{(Z_{ConRx} + Z_{ppRx} \parallel Z_L) \parallel Z_{GBRx} + Z_{retRx}}\right)\end{aligned} \tag{6}$$

Hence, $\eta_{Body-Rx}$ can be maximized by reducing Z_{ConRx}, Z_{retRx} and increasing Z_{ppRx}, Z_{GBRx}. The on-body signal strength in relation to the input voltage can expressed as follows:

$$\begin{aligned}V_{Rx}(\omega) = &\left(\frac{Z_{ppRx} \parallel Z_L}{Z_{ConRx} + Z_{ppRx} \parallel Z_L}\right) \cdot \\ &\left(\frac{(Z_{ConRx} + Z_{ppRx} \parallel Z_L) \parallel Z_{GBRx}}{(Z_{ConRx} + Z_{ppRx} \parallel Z_L) \parallel Z_{GBRx} + Z_{retRx}}\right) \cdot \eta_{Tx-Body} \cdot V_{Tx}(\omega)\end{aligned} \tag{7}$$

Supplementary Discussion 8: Influence of Tissue properties on Channel Characteristics

Consideration of the human body as a single-wire lossy transmission line may apparently introduce certain limitations: Specifically, the composition of the

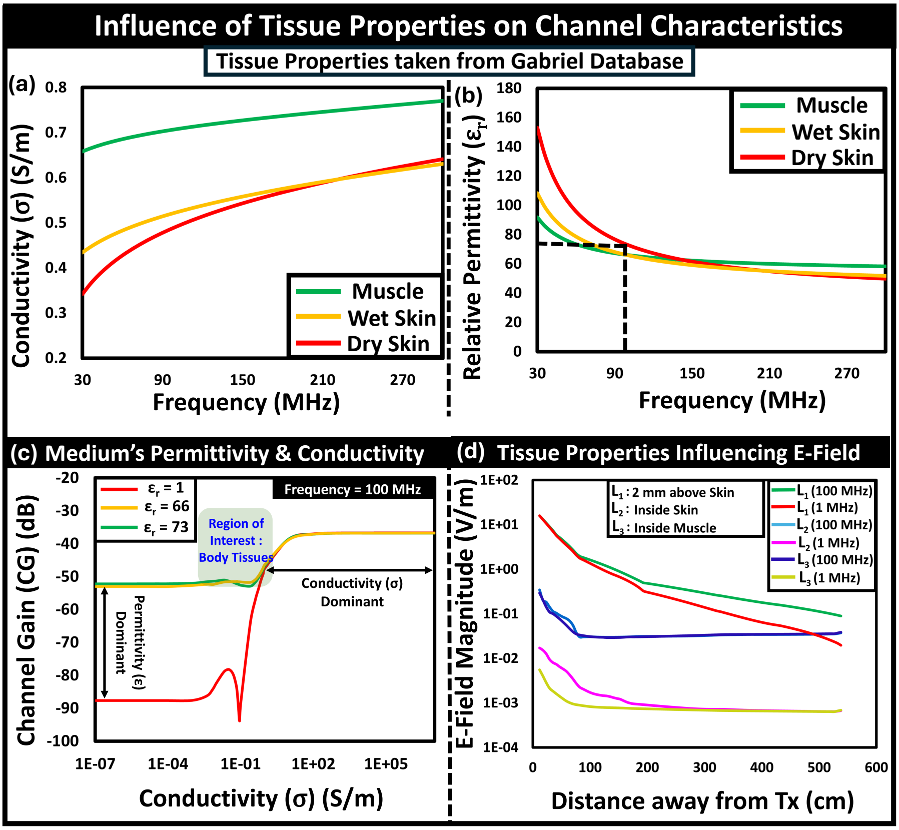

Supplementary Fig. 6 :Influence of Tissue Properties on Channel Characteristics: a. Conductivity of body tissues, **b.** Relative Permittivity of body tissues adopted from the work of Gabriel et al. **c.** Conductivity sweep, **d.** Comparison of Electric Field magnitude in different tissue layers in EQS (1 MHz) and BR (100 MHz).

body may seem to affect the body resonance phenomenon, raising questions about how to account for performance variations across different individuals. To address this concern, we conducted numerical simulations to analyze the variability of the BR HBC channel in terms of changes in body composition, specifically by varying relative permittivity ($\epsilon_r(f)$) and bulk conductivity ($\sigma(f)$) on a simplified model made up of muscle as the inner core with a 4 mm skin layer as the outer covering. The frequency-dependent variation of $\epsilon_r(f)$ and $\sigma(f)$ of Body Tissues (i.e., muscle, dry skin, and wet skin) in the BR frequency Regime obtained from the Gabriel database[5] is plotted in Supplementary Fig. 3 (a, b). In search of the underlying fundamentals, we artificially varied the ϵ_r and σ of body tissue. The variation in channel gain with change in conductivity for the relative permittivity of muscle, wet skin, and dry skin at different operating frequencies is presented in Supplementary Fig. 3 (c). Moreover, we have also observed variation in the magnitude of electric field from

the transmitter in three different scenarios, presented in Supplementary Fig. 6 (d): namely (a) 2 mm above skin, (b) Inside Skin, (c) Inside Muscle, we concluded that the E-Field magnitude remains higher above skin and decreases inside skin and goes on reducing even further inside muscle layer as it is more conductive than skin. In comparison, to EQS (operating frequency: 1 MHz), BR HBC (operating frequency: 100 MHz) experiences an increased magnitude of E-Field. From the results obtained, we conclude that the variability associated with the performance of the BR HBC channel remains within acceptable tolerance limits. With these variabilities within tolerance around the BR peak, the differences in channel gain across various subjects can be effectively managed by designing transceivers with user-specific adaptability. This implies that when users of this technology wear a BR HBC transmitter, it establishes a personalized connection (based on body parameters derived from data obtained through body-connected sensors and actuators) with BR HBC receivers (the user's body-connected devices) during the handshaking processes.

Supplementary Discussion 9: Communication Specificity Comparison of BR HBC with Radiative Wireless

Traditional radio frequency (RF)-based communication uses antennas at the transceivers (i.e., transmitter and receiver) to transfer information via radiation. These antennas, depending upon their polarization patten, incurs substantial off-body leakage as their principle of operation relies on broadcasting nature, hence causing security threats to users' personal information. For quantification, let's assume a RF antenna in the transmitting mode radiates a signal with power P_{Tx} and with antenna gain being G_{Tx}. The average radiated power density at a distance r from the antenna in free space can be approximated as:

$$P_D = P_{Tx} \cdot \frac{G_{Tx}}{4\pi r^2} \tag{8}$$

Owing to its conductivity and water content, the human body absorbs, reflects and scatters some fraction of the radiated signal, and the amount of signal loss depends on the tissue properties and the frequency. Now, for another antenna operating in the receiving mode, the Friis transmission equation, forms the basis for estimating its received power (P_{Rx1}) in free space which takes the following form:

$$P_{Rx1} = P_{Tx} \cdot G_{Tx} \cdot G_{Rx} \cdot \left(\frac{\lambda}{4\pi r_1}\right)^2 \tag{9}$$

Where, G_{Rx} = Gain of the receiving antenna, λ = Wavelength (c/f, where c is the speed of light and f is the operating frequency), r_1 = Distance between the transmitting and receiving antennas. For simplification, assuming line-of-sight scenario, the above expression P_{Rx1} gets modified in the presence of the human body to take the body's influence into account (i.e., loss factor α and

path-loss exponent n) and becomes:

$$P_{Rx1} = P_{Tx} \cdot G_{Tx} \cdot G_{Rx} \cdot \left(\frac{\lambda}{4\pi r_1}\right)^2 \frac{1}{(1+\alpha_1 r_1^{n_1})} \tag{10}$$

Assuming, a third antenna operating in receiving mode is located in the operational field of the RF-transmitter at a distance r_2 from the transmitting antenna but away from the human body intended to pick up the off-body leakage, the received power can be written as:

$$P_{Rx2} = P_{Tx} \cdot G_{Tx} \cdot G_{Rx} \cdot \left(\frac{\lambda}{4\pi r_2}\right)^2 \frac{1}{(1+\alpha_2 r_2^{n_2})} \tag{11}$$

Hence,

$$\frac{P_{Rx1}}{P_{Rx2}} = \frac{V_{Rx1}^2}{V_{Rx2}^2} = \frac{(1+\alpha_2 r_2^{n_2})}{(1+\alpha_1 r_1^{n_1})} \cdot \left(\frac{r_2}{r_1}\right)^2 \tag{12}$$

where, $\frac{V_{Rx1}}{V_{Rx2}} = \sqrt{\frac{P_{Rx1}}{P_{Rx2}}}$ represents the communication specificity (CS) as the ratio of on-body to off-body signal strength assuming iso sensitivity and iso-termination for the two receivers for a certain transmit power.

$$\text{CS} = \left(\frac{r_2}{r_1}\right) \cdot \sqrt{\frac{(1+\alpha_2 r_2^{n_2})}{(1+\alpha_1 r_1^{n_1})}} \tag{13}$$

under identical receiver distance from the transmitter (i.e., $r_1 = r_2 = r$),

$$\text{CS} = \sqrt{\frac{(1+\alpha_2 r^{n_2})}{(1+\alpha_1 r^{n_1})}} \tag{14}$$

In the context of wearables (size $\leq$ 3 cm) i.e., electrically small antennas, with Bluetooth operating within the 2.4 to 2.485 GHz frequency band, which corresponds to a wavelength (λ) of ~12.5 cm, making the field of observation (r $\geq$ 1 m) to be in the far field region ($r >> \max(\lambda\ , 2\text{D}^2/\lambda)$). With the Rx1 experiencing more attenuation from the body, i.e., $\alpha_1 >> \alpha_2$ and $n_1 > n_2$ the specificity factor reduces with comparable or higher off-body signal strength.

Now, for an iso-form factor, iso-sensitivity off-body voltage mode receiver, the received signal strength can be obtained as follows: since from the numerical simulations and measurements we observed that the Tx side experiences more leakage, hence we started our analysis with the leakage from a standalone BR HBC transmitter. The leakage signal strength from a standalone Tx can be expressed as:

$$V_{Leakage-Tx} = \frac{Z_{ppTx}}{Z_S + Z_{ppTx}} \cdot \frac{Z_{ppRx2} \parallel Z_{L2}}{Z_{ppRx2} \parallel Z_{L2} + Z_{Tx-Rx2} + Z_{retTx} + Z_{retRx2}} \cdot V_{Tx} \tag{15}$$

where Z_{L2} and Z_{retRx2} respectively represent the load impedance and the return path impedance of the off-body receiver, and Z_{Tx-Rx2} denotes the impedance between Tx and Rx2. Now, when the Rx2 is present closer to the human body in comparison to Tx, the leakage contribution from the human body can be expressed as:

$$V_{Leakage}(\omega) = \eta_{Tx-Body} \cdot \frac{Z_{ppRx2} \parallel Z_{L2} \parallel Z_{GBRx2}}{(Z_{ppRx2} \parallel Z_{L2}) \parallel Z_{GBRx2} + Z_{Body-Rx2} + Z_{retRx2}} \cdot V_{Tx}(\omega) \tag{16}$$

$Z_{Body-Rx2}$ denotes the impedance of the signal path between body and the off-body receiver. Hence for BR HBC, CS is defined as $V_{Rx}/V_{Leakage}$.

Supplementary Discussion 10: Regulatory constraints for BR HBC

The difference between intentional and unintentional radiators can be understood based on the operating frequency criteria as per the guidelines issued by FCC. In the BR frequency regime for frequencies between 30 and 88 MHz, if the electromagnetic fields measured at a distance of 3 meters are below 100 μV/m, and for frequencies from 88 to 216 MHz, if the fields at a distance of 3 meters are below 150 μV/m, the device may be classified as an unintentional radiator. This implies that no additional FCC certification is required for the operational deployment of these devices. In order to analyze the radiative component of BR HBC, we performed numerical simulations and experiments. Results from numerical simulation illustrate the electric field distribution from a human body with an active BR-HBC transmitter and the field decay characteristics over a distance away from the human body. In this context, we would like to highlight that due to its near-intermediate field operating range, the off-body electromagnetic field produced by an unshielded transmitter exceeds the FCC's specified standards when operating with an input excitation of 1 V amplitude, illustrated in Supplementary Fig. 7. To ensure compliance with FCC regulations, the following measures can be implemented: **(a)** Reduce transmit voltage by a factor of 10 and apply 5X duty cycling to the transmitted waveform, presented in Supplementary Fig. 7 (c). A 10X reduction in transmit voltage is expected to decrease the signal level by approximately 20 dB, which may negatively impact the signal-to-noise ratio (SNR) advantage of BR compared to EQS HBC. Nevertheless, BR HBC's atleast 5X higher bandwidth relative to EQS emphasizes the channel capacity benefits within the BR frequency range, which can be viewed as a broadband resonance that effectively combines the operational ranges of EQS and BR, presented in Supplementary Fig. 7 (b, c). **(b)** Another potential solution involves implementing a suitable shielding method or an efficient coupler design to ensure that the off-body field strength remains below the designated limits for unintentional radiators

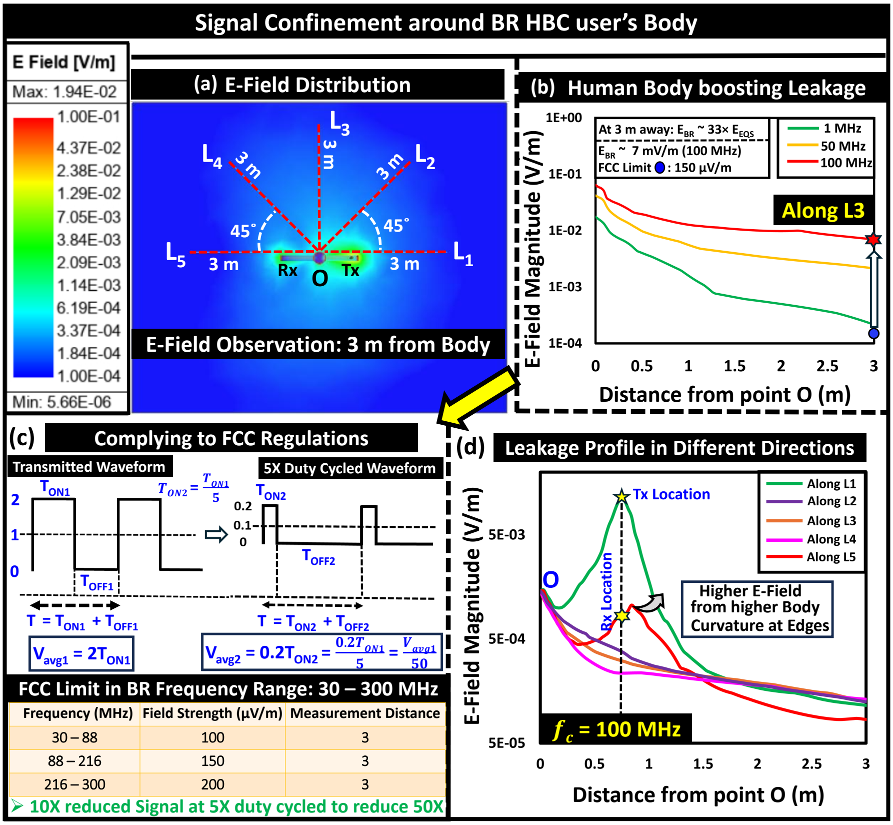

Supplementary Fig. 7 :**Leakage Profile Analysis: a.** E-Field Distribution near BR peak (100 MHz) illustrating the variation in E-Field from BR HBC user's body to ∼3 m, **b.** Variation in E-Field for different operating frequency EQS (1 MHz, 50 MHz, 100 MHz) depicting increased off-body signal strength at higher carrier frequency **c.** Reducing the average field strength by ∼50X (i.e., via 10X reduction in SNR and 5X duty cycling of the transmitted data) to comply with FCC regulations, **d.** Leakage Profile variations in different directions showing maximum leakage happens near the transmitter.

while maintaining a higher on-body SNR level. **(c)** New standards and certifications could be issued to deploy BR HBC. Despite these challenges, BR HBC's advantages, including its superior channel gain and capacity to support high data rates—continue to outweigh those of EQS HBC. Nonetheless, the advantages of BR HBC, such as its higher channel gain and ability to support high data rates, still surpass those of EQS HBC.

Supplementary Discussion 11: Comparison of BR HBC with prior related studies

The attributes of the prior studies are detailed below: Considering the human body as a monopole antenna, Kibret et al.[6] studied its antenna properties and its effect on human body communication over a frequency range of 1 MHz to

200 MHz. However, their use of a battery-powered vector network analyzer (VNA) and baluns for isolating the ground of the transmitter from the receiver, which may potentially lead to improper ground isolation, does not emulate the body channel measurements in the wearable-to-wearable scenario. Moreover, their use of galvanically coupled systems for on-body signal transmission analysis may raise concerns from a safety viewpoint. Using a similar approach, Li et al.[7] investigated wireless inter-human signal transmission for frequencies between 1 MHz and 90 MHz. Nevertheless, their use of wall-connected VNA does not take the parasitic coupling between the floating ground and the earth's ground (i.e., the return path capacitances of Tx and Rx) into consideration. Consequently, these approaches do not replicate measurements with wearable devices, thereby limiting the direct applicability of the insights gained from these studies.

Subsequently, Park et al.[8] presented path loss measurements for capacitive HBC using miniaturized battery-powered wearables and a moderate impedance matching network to maximize power transfer over a frequency range from 20 to 150 MHz. In their study on Body Channel Communication, Bae et al.[9] investigated how signals travel on the surface of the human body. They used a dipole model-based analysis to study components of the electric field, such as the near-field quasi-static coupling, reactive radiation, and the surface wave far-field across frequencies from 100 kHz to 100 MHz and distances of up to 1.3 m on the body. However, their methods, which involved using a wall-connected spectrum analyzer and balun, led to an optimistic estimation of the path loss and shifted the resonance peak of the human body to a lower frequency range, between 30 and 50 MHz. The experimental characterization of the body channel for capacitive HBC and its dependency on termination impedance using miniaturized wearable devices over a broad frequency range (100 kHz to 1 GHz) was done by Avlani et al.[10]. Their utilization of miniaturized devices with the floating ground though presents realistic path loss estimations, but the scope was limited as it does not analyze the factors influencing the channel variability. Additionally, Li et al.[11] demonstrated body-coupled power transmission and energy harvesting with wearable prototypes (Tx and Rx) in the frequency range of 30 MHz-90 MHz. However, the underlying theoretical framework for signal transmission in this frequency regime was not covered in this work.

While analyzing security and interference aspects of capacitive inter-body communication, a conceptual understanding of the body channel characteristics via identifying three regions, namely EQS, Body-Resonance, and Device Resonance, was proposed by Nath et al.[12] through wide-band measurements up to 1 GHz using wearable devices. They demonstrated that increasing the frequency beyond the EQS regime causes the human body to exhibit dimensional resonance between frequencies ranging from 60 MHz to 150 MHz as the operating wavelength approaches the human body's dimensions. Moving consequently higher in frequencies, such as in the range beyond 1 GHz, they observed device resonance that refers to the regime where the operating frequency is

high enough to make the wavelength comparable to the size of the devices, i.e., when capacitive HBC starts resembling RF-based communication with electrodes of the devices becoming antennas. Although this work has predicted the existence of the Body Resonance (BR) regime, where body dimensions become comparable to the wavelength, resulting in antenna resonance peaks, the precise locations of these peaks and their variability as well as the factors influencing their emergence in channel transfer characteristics—were beyond the scope of this study and warrant a more detailed investigation. In light of this research, recent attempts have focused on the impact of material properties of the signal transmission medium on body-resonance phenomena within 30 MHz to 300 MHz, as Sarkar et al.[13,14] provided insights based on wave impedance and illustrated their findings through measurements taken with wearable form factor devices. The key aspects are summarized in Table 1.

References

1. Datta, A., Nath, M., Yang, D., Sen, S.: Advanced biophysical model to capture channel variability for eqs capacitive hbc. IEEE Transactions on Biomedical Engineering (2021)

2. Modak, N., Das, D., Nath, M., Chatterjee, B., Kumar, G., Maity, S., Sen, S.: Eqs res-hbc: A 65-nm electro-quasistatic resonant 5–240 μw human whole-body powering and 2.19 μw communication soc with automatic maximum resonant power tracking. IEEE Journal of Solid-State Circuits **57**(3), 831–844 (2022)

3. Maity, S., Yang, D., Redford, S.S., Das, D., Chatterjee, B., Sen, S.: Bodywire-hci: Enabling new interaction modalities by communicating strictly during touch using electro-quasistatic human body communication. ACM Transactions on Computer-Human Interaction (TOCHI) **27**(6), 1–25 (2020)

4. Sarkar, S., Yang, D., Nath, M., Datta, A., Maity, S., Sen, S.: Human-structure and human-structure-human interaction in electro-quasistatic regime. Communications Engineering **4**(1), 26 (2025)

5. Gabriel et al., S.: The dielectric properties of biological tissues: II. measurements in the frequency range 10 hz to 20 GHz. Physics in Medicine and Biology **41**(11), 2251–2269 (1996). https://doi.org/10.1088/0031-9155/41/11/002

6. Kibret, B., Teshome, A.K., Lai, D.: Human body as antenna and its effect on human body communications. Progress In Electromagnetics Research **148**, 193–207 (2014)

Table 1 Comparison of proposed BR HBC with chronological evolution of high-speed wireless body-centric communication: This is the fundamental study that unveils the potential of BR HBC to support high-throughput wireless communications at ultra-low-power over on-body links with higher coverage

Author Approach	Operating Frequency(MHz)	Communication Devices (Tx & Rx) & Mode of Operation	Conceptual Channel Modeling & Theoretical Understanding	Link Type & Channel Variability	Signal Confinement Analysis & Tolerance to Interference
Bae et al.[9] Signals Propagation on surface of the human body	100 kHz-100 MHz	Wall-connected spectrum analyzer and isolating baluns leading to optimistic path loss estimation	No Channel model propagation mechanism in terms of near-field quasi-static coupling, reactive radiation, and the surface wave far-field	On-Body shifted resonance peak of the body to a lower frequency range, between 30-50 MHz with Ground connected devices	No
Kibret et al.[6] Body as a monopole antenna and its effect on HBC	1-200 MHz	battery-powered vector network analyzer (VNA) and baluns for ground isolation of devices Galvanic	No	On-Body Body as receiving antenna	No
Park et al.[8] Interactive Infrastructure via Body Channel Communication (BCC)	20-150 MHz	Miniaturized battery-powered wearables & impedance matching to maximize power transfer	No	On-Body	No
Li et al.[7] Inter-human signal transmission	1-90 MHz	wall-connected VNA i.e., optimistic path loss estimation with strong ground coupling	No	Inter-Body	No
Avlani et al.[10] Termination dependency of HBC channel	100 kHz-1 GHz	Wearable Tx & Rx Capacitive (E-Field based Pickup)	Simplified Bio-physical Model in EQS ($\leq$ 10 MHz) No model above EQS	On-Body No variability study	No
Nath et al.[12] Inter-body coupling	100 kHz–1 GHz	Wearable Tx & Rx Capacitive (E-Field based Pickup)	No Body as monopole antenna	On-Body (Intra-Body) Inter-Body No variability study	preliminary results of Inter-Body pickups are shown
Li et al.[11] Body-coupled power transmission and energy harvesting	30–90 MHz	Wearable Prototypes (Tx & Rx) Capacitive	Yes	On-Body (Intra-Body)	No
Sarkar et al.[14] Body Resonance Channel Measurements with Wearables	30-300 MHz	Wearable Tx & Rx Capacitive (E-Field based Pickup)	No	On-Body (variability with with Rx position) On-Body-to-Off-Body (distance variation)	No
Sarkar et al.[13] Material property based Surface wave Impedance-based understanding	30-300 MHz	Wearable Tx & Rx Capacitive (E-Field based Pickup)	No	On-Body No variability study	No
This work BR HBC	1-300 MHz (For Simulation) & 30-300 MHz (For Experiments)	Wearable Tx & Rx Capacitive (E-Field based Pickup)	On-Body (Intra-Body Channel) On-Body-to-Off-Body (Leakage Analysis) Inter-Body	Yes Conceptual model of Body as a lossy transmission line	Yes

7. Li, J., Nie, Z., Liu, Y., Wang, L., Hao, Y.: Evaluation of propagation characteristics using the human body as an antenna. Sensors **17**(12), 2878 (2017)

8. Park, J., Garudadri, H., Mercier, P.P.: Channel modeling of miniaturized battery-powered capacitive human body communication systems. IEEE Transactions on Biomedical Engineering **64**(2), 452–462 (2016)

9. Bae, J., Cho, H., Song, K., Lee, H., Yoo, H.-J.: The signal transmission mechanism on the surface of human body for body channel communication. IEEE Transactions on microwave theory and techniques **60**(3), 582–593 (2012)

10. Avlani, S., Nath, M., Maity, S., Sen, S.: A 100khz-1ghz termination-dependent human body communication channel measurement using miniaturized wearable devices. In: 2020 Design, Automation & Test in Europe Conference & Exhibition (DATE), pp. 650–653 (2020). IEEE

11. Li, J., Dong, Y., Park, J.H., Yoo, J.: Body-coupled power transmission and energy harvesting. Nature Electronics **4**(7), 530–538 (2021)

12. Nath, M., Maity, S., Avlani, S., Weigand, S., Sen, S.: Inter-body coupling in electro-quasistatic human body communication: Theory and analysis of security and interference properties. Scientific Reports **11**(1), 1–15 (2021)

13. Sarkar, S., Chowdhury, M.R., Huang, Q., Sen, S.: Material property based analysis of human body communication in body resonance regime. In: 2024 IEEE MTT-S International Microwave Biomedical Conference (IMBioC), pp. 69–71 (2024). IEEE

14. Sarkar, S., Huang, Q., Nath, M., Sen, S.: Wearable human body communication channel measurements in the body resonance regime. In: 2024 IEEE/MTT-S International Microwave Symposium-IMS 2024, pp. 800–803 (2024). IEEE

Acknowledgments:

This work was supported by Quasistatics, Inc. dba Ixana –Grant 40003567. The authors thank Lingke Ding, Meghna Roy Chowdhury, and David Yang, PhD students at Sparclab for their help and valuable input during the work.

Authors' contributions:

S. Sarkar, M. Nath, S. Sen conceived the idea. M.Nath was at Purdue University during his contribution to this work and also provided useful suggestions on the theory development and experiments thereafter. S. Sarkar and S. Sen

conducted the theoretical analysis. S. Sarkar conducted numerical simulations. S. Sarkar, S. Antal and Q. Huang performed the experiments. All the authors analyzed the results and reviewed the manuscript.

Competing interests:

The authors declare that S. Sen have a financial interest in Quasistatics, Inc. and the remaining authors declare no competing interests.

Additional information:

Additional supplementary information is available at https://github.com/SparcLab/BodyResonanceHBC. Correspondence and requests for materials should be addressed to Shreyas Sen (shreyas@purdue.edu) or Samyadip Sarkar (sarkar46@purdue.edu).
