# Supplementary material for: Body-resonance: transmission line-like wireless links enabling high-speed wearable communication
Source: Commun Eng. 2025 Dec 20;5:1. doi: 10.1038/s44172-025-00533-z (PMC12764851; doi:10.1038/s44172-025-00533-z)
Supplement: Supplementary file 2 — Description of Additional Supplementary Files [file 44172_2025_533_MOESM2_ESM.pdf]

Description of Additional Supplementary Files

File name: Supplementary Movie 1

Description: We present how the magnitude of the electric field and the Poynting vector vary with changes in operating frequency around a Human Body Model. The variation in the electric field highlights the advantages of Body Resonance Human Body Communication, in terms of enhanced channel capacity, where the body acts as a transmission line, in contrast to Electro-Quasistatic Human Body Communication, where the body behaves like a wire. Additionally, we illustrate the equipotential nature of the human body in Electro-Quasistatics, which transitions to the formation of electromagnetic resonant patterns in the Body-Resonance regime. The variation in the Poynting vector demonstrates the guided nature of energy flux density around the body, confirming the reduced leakage of Body Resonance Human Body Communication compared to traditional radiative antenna-based wireless communications.

File name: Supplementary Movie 2

Description: We present the variation in the magnetic field as the operating frequency changes around a Human Body Model. This reveals the formation of magnetic (H) loops that encircle the body and return to the ground, with the field's magnitude increasing in the Body Resonance frequency range. The observed pattern of H loops can be considered the magnetic signature of a guided transmission line connecting the cross-cylindrical human body model and the Earth's ground plane.

File name: Supplementary Movie 3

Description: We present the variation in the magnitude of the electric field as the phase of the applied AC excitation changes. This phase change can be correlated with the transient response, allowing us to capture the temporal variation of the field magnitude.
