# Supplementary material for: Body-resonance: transmission line-like wireless links enabling high-speed wearable communication
Source: Commun Eng. 2025 Dec 20;5:1. doi: 10.1038/s44172-025-00533-z (PMC12764851; doi:10.1038/s44172-025-00533-z)
Supplement: Supplementary file 6 — Reporting Summary [file 44172_2025_533_MOESM6_ESM.pdf]

nature portfolio

Corresponding author(s): Shreyas Sen (shreyas@purdue.edu)

Last updated by author(s): Aug 27, 2025

Reporting Summary

Nature Portfolio wishes to improve the reproducibility of the work that we publish. This form provides structure for consistency and transparency in reporting. For further information on Nature Portfolio policies, see our Editorial Policies and the Editorial Policy Checklist.

Statistics

For all statistical analyses, confirm that the following items are present in the figure legend, table legend, main text, or Methods section.

n/a	Confirmed	
☐	☒	The exact sample size (*n*) for each experimental group/condition, given as a discrete number and unit of measurement
☐	☒	A statement on whether measurements were taken from distinct samples or whether the same sample was measured repeatedly
☒	☐	The statistical test(s) used AND whether they are one- or two-sided<br>*Only common tests should be described solely by name; describe more complex techniques in the Methods section.*
☒	☐	A description of all covariates tested
☒	☐	A description of any assumptions or corrections, such as tests of normality and adjustment for multiple comparisons
☐	☒	A full description of the statistical parameters including central tendency (e.g. means) or other basic estimates (e.g. regression coefficient) AND variation (e.g. standard deviation) or associated estimates of uncertainty (e.g. confidence intervals)
☒	☐	For null hypothesis testing, the test statistic (e.g. *F*, *t*, *r*) with confidence intervals, effect sizes, degrees of freedom and *P* value noted<br>*Give P values as exact values whenever suitable.*
☒	☐	For Bayesian analysis, information on the choice of priors and Markov chain Monte Carlo settings
☒	☐	For hierarchical and complex designs, identification of the appropriate level for tests and full reporting of outcomes
☒	☐	Estimates of effect sizes (e.g. Cohen's *d*, Pearson's *r*), indicating how they were calculated

Our web collection on statistics for biologists contains articles on many of the points above.

Software and code

Policy information about availability of computer code

Data collection	This following software/tools were used in this study for the numerical electromagnetic simulation: HFSS (Ansys, version 2023 R1, version 2023 R2)
Data analysis	No custom data analysis was performed. The plots of the results from the numerical simulation and experiments were generated with Microsoft Excel (Version 2021).

For manuscripts utilizing custom algorithms or software that are central to the research but not yet described in published literature, software must be made available to editors and reviewers. We strongly encourage code deposition in a community repository (e.g. GitHub). See the Nature Portfolio guidelines for submitting code & software for further information.

Data

Policy information about availability of data

All manuscripts must include a data availability statement. This statement should provide the following information, where applicable:

- Accession codes, unique identifiers, or web links for publicly available datasets
- A description of any restrictions on data availability
- For clinical datasets or third party data, please ensure that the statement adheres to our policy

The data, supporting the plots and other findings are provided in this paper and its supplementary document. The supplementary document is available at the

provided github link: https://github.com/SparcLab/BodyResonanceHBC.
Any other materials on the plots are available from the corresponding author upon reasonable request.

Research involving human participants, their data, or biological material

Policy information about studies with human participants or human data. See also policy information about sex, gender (identity/presentation), and sexual orientation and race, ethnicity and racism.

Reporting on sex and gender	This research focuses on the scientific and system validation of Body Resonance Human Body Communication and collection of data from human with different sex and gender was not needed and/or performed.
Reporting on race, ethnicity, or other socially relevant groupings	This research focuses on the scientific and system validation of Body Resonance Human Body Communication and collection of data from human with different race, ethnicity, or other socially relevant groupings was not needed and/or performed.
Population characteristics	The measurement of performance of Body Resonance Human Body Communication were executed with subjects (3 males), with heights ranging from 165 cm to 180 cm, weighing between 60 kg to 80 kg and aged between 22 and 28
Recruitment	After obtaining informed consent, subjects were recruited from within the research group from Purdue University
Ethics oversight	The experiments involving human subjects were conducted while complying with all the guidelines and regulations given by the Purdue Institutional Review Board (IRB Protocol 1610018370).

Note that full information on the approval of the study protocol must also be provided in the manuscript.

Field-specific reporting

Please select the one below that is the best fit for your research. If you are not sure, read the appropriate sections before making your selection.

☒ Life sciences ☐ Behavioural & social sciences ☐ Ecological, evolutionary & environmental sciences

For a reference copy of the document with all sections, see nature.com/documents/nr-reporting-summary-flat.pdf

Life sciences study design

All studies must disclose on these points even when the disclosure is negative.

Sample size	The central focus of this research being the validation of the communication system for Body Resonance Human Body Communication, large number of biological samples/animals were not used. The measurements were performed on 3 human subjects (3 males), with heights ranging from 165 cm to 180 cm, weighing between 60 kg to 80 kg and aged between 22 and 28. The statistical significance of measured datasets by obtaining consistent data points while repeating each experiment over several weeks. The experimental datasets are plotted for the nominal and repeatable scenarios with statistical conformity provided in terms of plotting the mean value of the data points and standard deviation (shown with the error bars).
Data exclusions	The collected data from the nominal and repeatable scenarios are presented while excluding the non-repeatable measurements.
Replication	The measurements on human subjects were conducted over several days and the replication of the result was successful.
Randomization	This research focuses on the scientific and system validation of Body Resonance Human Body Communication and no randomization studies were not needed and/or performed.
Blinding	This research focuses on the scientific and system validation of Body Resonance Human Body Communication and no blinding studies were not needed and/or performed.

Reporting for specific materials, systems and methods

We require information from authors about some types of materials, experimental systems and methods used in many studies. Here, indicate whether each material, system or method listed is relevant to your study. If you are not sure if a list item applies to your research, read the appropriate section before selecting a response.

Materials & experimental systems

n/a	Involved in the study
☒	☐ Antibodies
☒	☐ Eukaryotic cell lines
☒	☐ Palaeontology and archaeology
☒	☐ Animals and other organisms
☒	☐ Clinical data
☒	☐ Dual use research of concern
☒	☐ Plants

Methods

n/a	Involved in the study
☒	☐ ChIP-seq
☒	☐ Flow cytometry
☒	☐ MRI-based neuroimaging

Plants

Seed stocks	N/A, No plant studies were performed.
Novel plant genotypes	N/A, No plant studies were performed.
Authentication	N/A, No plant studies were performed.
